# Understanding school-based rehabilitation services through the lived experiences of children and youth with disabilities: a meta-aggregative review

**DOI:** 10.3389/fpubh.2026.1745224

**Published:** 2026-02-27

**Authors:** Amelia Brushett, Kelsey Seguin, Logan Wong, Clarissa McCarry-Taillefer, Peter Rosenbaum, Tara Packham, Wenonah Campbell

**Affiliations:** 1 School of Rehabilitation Science, McMaster University, Hamilton, ON, Canada; 2 CanChild Centre for Childhood Disability Research, McMaster University, Hamilton, ON, Canada; 3 School of Rehabilitation Science, University of Ottawa, Ottawa, ON, Canada; 4 Faculty of Arts and Science, Concordia University, Montreal, QC, Canada; 5 Department of Pediatrics, McMaster University, Hamilton, ON, Canada

**Keywords:** disability, inclusion, occupational therapy, participation, physiotherapy, school-based rehabilitation services, speech-language pathology, youth engagement

## Abstract

Children and youth with disabilities continue to face barriers to accessing quality education, despite education being a key social determinant of health. School-based rehabilitation services (SBRS) include occupational therapy (OT), physiotherapy (PT), and speech-language pathology (SLP) that are delivered within the school context to address these barriers by promoting participation and inclusion. However, the implementation of SBRS has largely prioritized adult perspectives, with limited consideration of the lived experiences of children and youth with disabilities. Guided by the Joanna Briggs Institute (JBI) guidelines for meta-aggregation, this review synthesizes primary qualitative studies exploring the lived experiences of children and youth with disabilities who receive SBRS. Following a systematic selection process and critical appraisal, 13 studies were included. A total of 53 findings were extracted, grouped into 14 categories, and synthesized into six overarching findings. Specifically, children and youth reported that they want (i) therapists to increase their autonomy and agency by clearly communicating the purpose and goals of therapy and supporting their ability to make informed choices; (ii) therapists to adopt a holistic approach by addressing both social-emotional and functional needs; (iii) therapists who are knowledgeable, supportive, empathetic, and who advocate for their needs; (iv) therapy that is individualized, meaningful, enjoyable, and scheduled in a way that respects school routines, enhancing their participation in both therapy and broader school life; (v) therapy to help them build skills supporting social connection and inclusion at school; and (vi) therapy in which they set goals and develop new skills. Findings showed children and youth with disabilities want to be involved in their therapy and expect therapists to equip them with skills to participate, make decisions, and be included at school and in therapy. The findings have important implications for SBRS practice and policy, highlighting the importance of centering the voices of children and youth with disabilities. Attending to children's and youths' perspectives can help foster practices and policies that are inclusive, holistic, and child-centered, and supports the development of services that are meaningful, empowering, and promote full participation in school life.

## 1 Introduction

Disability affects over 240 million children and youth worldwide, and these numbers continue to rise (1). In 2021, UNICEF estimated that approximately one in ten children and youth globally live with a disability. As the population of children and youth with disabilities grows, it becomes increasingly important to examine whether education systems are adequately addressing their rights and needs (2).

Education is a fundamental right for all children. Articles 23 and 28 of the United Nations Convention on the Rights of the Child (UNCRC) affirm that all children, including those with disabilities, are entitled to access quality education (3). Despite this entitlement, children and youth with disabilities remain among the most vulnerable groups within education systems, with many of their educational needs unmet by current systems (2, 4). This reality highlights the urgent need to critically examine how education systems can better support equitable access to quality education for all children and youth.

To conceptualize disability and functioning, the World Health Organization's (WHO) International Classification of Functioning, Disability and Health (ICF) offers a widely recognized framework that views disability as a dynamic interaction between individuals and their environments (5). Within the ICF, functioning encompasses body structures, body functions, activities, and participation, whereas disability involves impairments, activity limitations, and participation restrictions. The ICF recognizes that functioning and disability are closely interlinked and are influenced by an individual's environment (5).

Participation is a key concept of the ICF and is defined as “involvement in life situations” [(5), p. 12]. In school contexts, participation includes engagement in both structured activities, such as classroom learning and extracurricular programs, and unstructured activities, such as recess, playtime, and social interactions (6). Participation in school activities is crucial to childhood development, as it fosters identity, autonomy, skill-building, and social connection, and is linked to positive social, emotional, academic, and health outcomes in children and youth (7–13).

The positive effects of school participation and its role in childhood development are well documented (7–13). For example, a study by de Róiste et al. (8) found that genuine participation was associated with higher academic achievement, greater enjoyment of school, and increased happiness during the school day. However, despite these benefits, research consistently indicates that children and youth with disabilities participate less frequently in school activities than their non-disabled peers (14–16). These disparities can be attributed to systemic barriers, including inaccessible environments, inadequate accommodations, underfunded support services, and negative societal attitudes (16–18). Ensuring that children can not only access school but participate meaningfully requires policy changes and ongoing examination of the structures and services that support children and youth at school.

The WHO describes rehabilitation services, such as occupational therapy (OT), physiotherapy (PT), and speech-language pathology (SLP) as services aiming to support individuals' participation in everyday activities and occupations, including school, work, and recreation (19). Rehabilitation services are often implemented directly in schools to support participation of children and youth in school activities (9, 20, 21). In this review, the term school-based rehabilitation services (SBRS) is used as an umbrella term to describe OT, PT, and SLP services delivered within school settings (21).

Terminology and service delivery models of rehabilitation services vary internationally. For example, similar services are referred to as school-based allied health interventions in the United Kingdom (9) and as related services in the United States (20). Although there are variations across contexts, these services share common goals of fostering inclusion and participation by addressing functional, environmental, physical, and communication needs within the school context (9, 21–25).

In addition to SBRS, inclusive education has emerged globally as a key approach to addressing inequities in access to education (26–28). It emphasizes educating all students together in mainstream settings, regardless of ability, background, or learning needs (29). Inclusive education shifts responsibility away from the child and toward the education system, recognizing that systems must be transformed to support diversity (29). This approach is reinforced by global frameworks such as the Salamanca Statement and Framework for Action (30) and the UNCRC (3), which affirm the right of all children to inclusive, quality education (26, 31, 32).

Despite these global commitments and implementation of supports, many children and youth with disabilities continue to face challenges in practice. Persistent inequities in educational outcomes, peer relationships, and school engagement remain evident worldwide (1, 4).

While SBRS are designed to support inclusion and participation of children and youth, research and service design have largely centered adult perspectives, including those of teachers, therapists, and parents, leaving the voices of children and youth underrepresented (33–39). This adult-centered focus contradicts the recognition that children and youth are experts in their own lives and fails to uphold the commitments of Article 12 of the UNCRC, which affirms children's right to express their views in all matters affecting them and requires that these views be given due weight according to age and maturity (3). Accordingly, children's and youths' perspectives are essential to informing research, service delivery, and policy development of SBRS (35, 39–41).

Although there has been a shift toward conducting research *with* children rather than *on* children, significant gaps remain in how children's voices are meaningfully integrated in research (38, 42–44). For example, Njelesani et al. (44) found that despite a growing number of qualitative studies involving children with disabilities, children's voices often remain passive and are not actively used to inform research. In addition, while qualitative research exploring the experiences of children and youth with disabilities is growing, findings remain fragmented and have not been synthesized in ways that amplify children's voices across studies (44).

To address this gap, we utilized the Joanna Briggs Institute (JBI) guidelines (45) for meta-aggregation to collect and synthesize existing literature on the experiences of children and youth with disabilities receiving SBRS. This review had two aims: (1) to understand what children and youth with disabilities want and need from their SBRS, and (2) to develop practical, actionable recommendations for policies and practice.

By synthesizing qualitative studies, this review amplifies the voices of children and youth with disabilities and aims to inform service delivery while providing evidence to support child-informed practice, policy development, and decision-making within SBRS policies and practices.

## 2 Methods

### 2.1 Research question and search strategy

Our meta-aggregative review (45) addressed the following question: What are the lived experiences of children and youth with disabilities regarding the SBRS they receive? The protocol was registered on the Open Science Framework (https://doi.org/10.17605/OSF.IO/BM48C).

The review question and search strategy were developed with guidance from the PICo framework (Population, Interest, Context) (45). The population included children and youth from school entry to school exit eligible for SBRS. Our focus was on their lived experiences and perspectives, specifically related to OT, PT, and SLP services provided in school settings. The context encompassed both publicly and privately funded services provided in mainstream and specialized schools.

The search strategy was developed in collaboration with a health sciences librarian at McMaster University. Using PICo, we identified subject headings and keywords related to our population, lived experiences and qualitative research, rehabilitation, and school settings. Terms within each concept were combined with “OR” and then the different concepts were connected with “AND.” The final strategy was refined in consultation with the librarian to ensure it was comprehensive and appropriately adapted for each database searched.

Searches were conducted in six databases: CINAHL, Embase, Emcare, ERIC, Medline, and Web of Science. Table 1 provides examples of search terms and the structure of search strategies. Complete search strategies for the six database searches can be found in Appendix A.

**Table 1:** Example of search terms and search strategy.

**Search concept**	**Population (children and youth)**	**Interest (perspectives and qualitative terms)**	**Interest (rehabilitation)**	**Context (school)**
Key terms	(Child^*^ OR youth^*^)	(Lived experience^*^OR perspective^*^)	(Occupational therap^*^ OR physiotherap^*^)	(School^*^ OR school base^*^)

### 2.2 Qualitative evidence synthesis: meta-aggregation

Meta-aggregation is an approach to aggregating evidence from existing qualitative research to inform policy and practice (45, 46). Rooted in pragmatism, it balances the importance of meaningfully representing human lived experience (47) with the need to produce actionable, evidence-based knowledge to inform decision-making in real-world contexts (45–48). Meta-aggregation is well suited to the aims of this review, which seeks to amplify the voices of children and youth with disabilities to inform SBRS policy and practice.

In contrast to interpretive synthesis approaches such as meta-ethnography, which aim to re-interpret data and generate new theoretical insights across studies, meta-aggregation involves a synthesis that remains close to the original interpretations of the authors and aggregates findings without re-conceptualization (45, 49). Avoiding reinterpretation of the original findings was essential for our review, as we aimed to authentically represent how children and youth with disabilities describe their lived experiences with SBRS.

### 2.3 Eligibility criteria

This review included primary qualitative and mixed methods studies that employed research methods such as interviews, focus groups, and other qualitative approaches to explore the lived experiences and perspectives of children and youth eligible for SBRS. Methodologies of interest included studies that utilized phenomenology, grounded theory, and other qualitative methodologies. Quantitative studies were excluded. Only primary research was included, while secondary analyses such as systematic reviews, meta-analyses, and scoping reviews were excluded. Eligible literature consisted of scholarly sources, including research articles, dissertations, theses, and book chapters. Government documents, conference proceedings, and other non-scholarly literature were excluded.

The population of interest was children and youth with disabilities. We included studies that collected lived experiences and perspectives from children and youth from school entry to school exit. Perspectives from adults recounting their school-aged experiences were also included. Studies focusing on individuals in preschool, post-secondary education, or on non-student perspectives such as parents, educators, or healthcare providers were excluded. We included studies that collected lived experiences and perspectives from adult participants alongside those of our population of interest. However, studies were included only if the perspectives of children and youth were clearly distinguishable from those of adult participants (e.g., participant quotations were labeled such that it was clear whose perspectives were represented). This distinction allowed us to extract themes and illustrative data specific to children and youth. Only first-person perspectives were included to ensure the review captured the lived experiences and voices of children and youth themselves.

The context of the review was limited to services provided in school environments, including both publicly and privately funded schools, whether in mainstream or specialized settings. Studies examining rehabilitation services delivered outside of school settings were excluded, as were those focusing on preschools, daycares, before or after school programs, or post-secondary institutions. Eligible services were limited to OT, PT, and SLP services provided within the defined school context. Studies examining other types of services, such as psychology, nursing, social work, or dietetics, were excluded. A list of eligibility criteria can be found in Appendix B.

### 2.4 Research approach and review team

This review employed an Integrated Knowledge Translation (iKT) framework to engage youth with disabilities actively in the research process. The iKT framework emphasizes the co-production of knowledge through close collaboration between researchers and knowledge users across all stages of research (50). While participatory approaches, such as youth participatory action research (YPAR), provide important models for engaging youth as co-researchers, YPAR is primarily oriented toward action-based, primary research (51, 52). An iKT framework is designed to involve knowledge users across all stages of research to enhance the relevance and applicability of findings for policy and practice (50). Therefore, iKT was selected for its alignment with the review methodology and aims (50, 53).

Our review team included five individuals: primary reviewer (AB) and secondary reviewers (CMT, IK, KS, LW), three of whom (CMT, KS, LW) identify as having disabilities and have lived experiences with rehabilitation services. The members of the review team with lived experience (CMT, KS, LW) were recruited through Canada's Child-Bright Network's Training and Capacity Building Program, which aims to foster patient-oriented research by engaging partners with lived and living experience (54). Individuals who expressed interest in participating contacted the external reviewer (WC) to indicate their interest in being involved.

Following their expression of interest, review team members with lived experience were hired as research team members through McMaster University. As part of this process, team members completed standard institutional training requirements, including university-mandated health and safety modules. Although team members had varied prior experience with research ethics, no formal ethics training was provided.

Consistent with the iKT framework, the review team members with lived experience played a key role in shaping research decisions and ensuring that the perspectives of individuals with disabilities were meaningfully integrated throughout the review, from protocol development to dissemination (52). An additional member of the review team (IK) joined after the search was completed and was recruited through the external reviewer's network of research assistants. IK contributed to the screening, critical appraisal, and data extraction stages. However, unlike the review team members with lived experience, IK did not participate in advising on research decisions or data synthesis.

To establish clear roles, tasks, and responsibilities, the team used the Involvement Matrix (55). This tool supported effective communication between the secondary review team members and the primary reviewer, allowing them to specify where they wished to be most involved and to set clear expectations and roles throughout the review.

Prior to commencing the review, all review team members met with the primary reviewer to orient to the project's aims, methodology, and expectations. Review team members received tailored training related to qualitative research methods, systematic review processes, and the principles of meta-aggregation. These sessions were designed to support meaningful participation and research skill development and provided opportunities for secondary reviewers to indicate their preferred level of involvement at each stage of the review and to identify any accommodations required.

Before each stage of the review, review team members participated in group meetings, with additional individual meetings offered as needed to support skill development and understanding of each task. The primary reviewer developed and circulated accessible training materials, including written guides, examples, and step-by-step instructions, to support consistency and confidence across the team.

### 2.5 Selection of studies

The review team conducted the screening process in two stages: title and abstract screening followed by full-text review, using Covidence software. Automatic deduplication was performed within Covidence prior to the title and abstract screening, and manual deduplication was conducted during screening to remove any duplicates missed by the software.

The primary reviewer facilitated two initial training sessions to establish reliability among team members. In the first session, all reviewers independently screened 10 titles and abstracts, and in the second session, an additional 10 titles and abstracts were reviewed. After each session, the team convened to compare decisions, resolve conflicts through discussion, and refine the eligibility criteria. Following the training, inter-rater reliability testing was conducted using 75 articles. The team reached a consensus level of 80% or higher before commencing the formal title and abstract screening phase. An 80% agreement threshold was selected, as agreement at or above this level reflects a strong level of agreement between raters (56, 57).

During the title and abstract screening, the primary reviewer met regularly with the secondary reviewers to address conflicts. Conflicts were resolved through discussion and consensus. When consensus could not be reached, an external reviewer was consulted to make a final decision. Any sources that did not have an abstract available for screening automatically advanced to full-text review.

Following the title and abstract screening, the same five reviewers proceeded to full-text review. Before starting this phase in Covidence, the primary reviewer led a training session where the team reviewed five full-text articles. The review team then met to discuss inclusion and exclusion decisions and further refine the eligibility criteria. Inter-rater reliability testing was conducted using 10 full-text articles, achieving an agreement level of 80% or higher between the primary reviewer and each secondary reviewer. Once this reliability threshold was met for each team member, the team completed full-text review in Covidence. Any disagreements were resolved by consensus; if consensus between the reviewers could not be reached, an external reviewer was consulted to make a final decision.

### 2.6 Critical appraisal

As outlined in the JBI Manual for Evidence Synthesis (45), because the findings of a meta-aggregative review are intended to inform policy and practice, critical appraisal is a necessary step for ensuring selection of high-quality studies (45).

For this step, the review team utilized a modified standardized JBI critical appraisal checklist (58). This modified checklist was created in consultation with the JBI and experienced qualitative researchers to enhance its use by novice researchers (58). The modifications included incorporating explanations and examples alongside critical appraisal criteria to guide decision-making (58). Given that all members of the review team were novice researchers, the added guidance enabled the team to critically assess with confidence whether articles met the pre-established quality criteria.

The critical appraisal checklist included two screening questions that assessed relevance of included articles to the review question. If a study did not pass both screening questions, it was excluded. Studies deemed relevant by both screening questions were further appraised for methodological quality using seven additional questions. The critical appraisal checklist is presented in Appendix C.

The same reviewers who conducted the screening also participated in a training session for critical appraisal led by the primary reviewer. Following the training session, secondary reviewers applied their training by independently appraising one study as a team and one study independently. Each reviewer then met with the primary reviewer to discuss their appraisal and decisions. Once all reviewers demonstrated understanding of the critical appraisal process, secondary reviewers were assigned specific studies to appraise. All articles were appraised by the primary reviewer and one of the four secondary reviewers. The primary reviewer met regularly with the secondary reviewers individually to discuss decisions and resolve any disagreements. In cases where consensus could not be reached, an external reviewer was consulted.

### 2.7 Data extraction

The studies included after the critical appraisal stage were extracted by the review team in two phases. In the first phase, the review team extracted the main study characteristics and relevant information to the research question (see Table 2). These data were extracted by the primary reviewer and one other member of the review team using Covidence. Covidence flagged any conflicts, which were then resolved through discussion and consensus between the primary reviewer and the second reviewer.

**Table 2:** Data extraction template for main study characteristics.

**Study identification**	**Study focus**	**Study methods**			**Participant characteristics**			**Setting**
Authors and date	Phenomenon of interest	Methodology	Method of data collection	Data analysis approach	Participant age and gender	Disability	SBRS of interest	Location of study

In the second phase, the primary reviewer extracted relevant findings from the included studies. Per the JBI Manual for Evidence Synthesis (45), a finding was defined as “a verbatim extract of the author's analytic interpretation of their results or “data”” [(45), p. 67]. Each finding was accompanied by extracted illustrations, defined as “a direct quotation of a participant's voice” [(45), p. 67].

A verbatim description of each theme or category was extracted, with sub-themes extracted separately when applicable. Additionally, positive and negative perspectives within themes were extracted as separate findings, as they would be classified differently during synthesis. When descriptions were lengthy or dispersed across multiple sections of the text, the primary reviewer paraphrased the description while maintaining the original meaning and interpretation of the authors. The first supporting illustration was extracted for each finding, with additional illustrations extracted when a single illustration did not adequately capture the complexity or full scope of the theme.

All extracted findings were verified by a secondary reviewer, who assessed the accuracy, completeness, and appropriateness of the extracted descriptions and illustrations.

Following extraction, each finding was assigned a level of credibility using the definitions provided in the JBI Manual for Evidence Synthesis (45). A finding was classified as *unequivocal* when it was supported by a participant illustration that clearly and unambiguously aligned with the author's interpretation. Findings were classified as *credible* when the illustration was logically consistent with the finding but open to challenge or alternative interpretation. Findings were classified as *unsupported* when no illustration was provided or when the illustration did not clearly support the stated finding.

Both the primary reviewer and a secondary reviewer independently assigned a level of credibility to each finding while blinded to one another's assessments. The primary reviewer then compared assigned credibility levels, and any conflicts were resolved through discussion and consensus. See Table 3 for the extraction template for study findings and examples of how findings were extracted, and credibility rating assigned.

**Table 3:** Data extraction template for findings with credibility examples.

**Extracted theme/category/title**	**Description (verbatim description from authors)**	**Illustration direct quote/s from participants**	**Level of credibility according to JBI guidelines**
	**Location specified as page #, column #, paragraph #, lines #-#**	**Location specified as page #, column #, paragraph #, lines #-#**	
Example: theme related to school participation and accommodations (70)	Participation in class continued to be a concern for some students who stutter, and some will try and avoid speaking situations. Some students benefited from accommodations, which were specific to each student (page 1332, column 2, paragraph 1, lines 1-4)	Participant 7: “I try to avoid public speaking. When I present, most of my teachers let me go after school when the students are not there. I don't do it all of the time because sometimes I have the courage to go up and do it. If I feel like I can do it, then I want to do it. When I am reading in class, my teacher lets me go up and read in private. My speech therapist at school when I had a presentation in my English class, she talked with my English teacher and said that if I could pick a couple of friends to come and listen to the presentation. Maybe not all of them but I do remember most of them being helpful toward anything.” (page 1332, column 2, Quote 7)	Reviewer 1: unequivocal Reviewer 2: unequivocal
Create safe school environments: educating others (66)	Participants emphasized the need for adults in schools to understand stuttering and provide support. They believed all staff should be educated on the variability of stuttering, the loss of control it involves, and why advice like slow down or take a breath is unhelpful. Educating school staff, parents, and peers was seen as crucial, with suggestions that speech therapists provide training in meetings or one-on-one discussions. Classroom presentations were also recommended to help peers understand stuttering, but participants stressed that these should be child-driven, allowing the child to decide if and how they want to share their experiences. While presentations could be beneficial, they could also be harmful if not handled with care and without ensuring the child's autonomy in the process. (paraphrased-page 107, column 2, paragraph 2–3)	“[showing] there are other people who know and who get it, who [they] can find support in. I think that would be really big” (Felix Felipe) (page 107, column 2, paragraph 2, lines 10-13)	Reviewer 1: credible Reviewer 2: credible

### 2.8 Data synthesis

After data extraction, the findings were synthesized by the primary reviewer and verified by review team members with lived experience, as well as the external reviewer. Following the JBI Manual for Evidence Synthesis (45), the primary reviewer organized the findings into categories. JBI defines a category as “a brief description of a key concept arising from the aggregation of two or more like findings and is accompanied by an explanatory statement that conveys the whole, inclusive meaning of a group of similar findings” [(45), p. 67]. These categories were then presented to the members of the review team with lived experience for verification. During the meeting, the review team members with lived experience provided input and guidance on the category names and descriptions, as well as suggestions for additional categories. The primary reviewer then met with the external reviewer to refine the categories based on the input from the review team and provide any additional input on the categories.

After refining the categories with input from both the review team members with lived experience and the external reviewer, the primary reviewer re-presented the categories to both groups for verification. At this time, all review team members with lived experience endorsed the categories. The primary reviewer then grouped the categories into synthesized findings. JBI defines a synthesized finding as “an overarching description of a group of categorized findings” [(45), p. 67]. “Synthesized findings are expressed as “indicatory” statements that can be used to generate recommendations for policy or practice” [(45), p. 67]. Like categories, each synthesized finding is accompanied by an explanatory statement that conveys the meaning of the grouped categories.

After generating the synthesized findings, the primary reviewer followed the same approach to synthesizing categories. The primary reviewer met with the review team with lived experience for feedback and suggestions and then refined the synthesized findings with the external reviewer. After all statements were finalized, review team members with lived experience were consulted for their final opinions.

**Figure 1:**
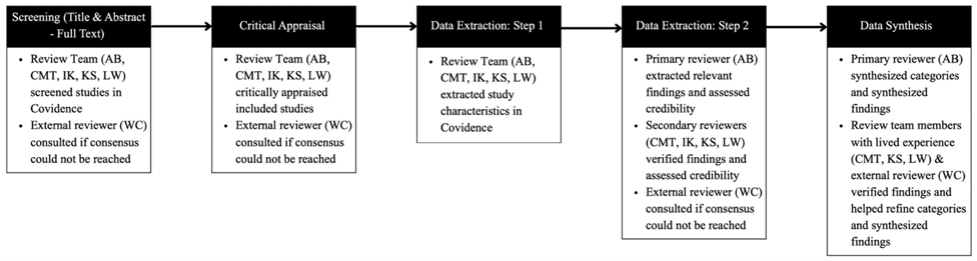
Review process and review team responsibilities.

The review process and roles of reviewers are outlined in Figure 1.

## 3 Results

After conducting the search across six relevant databases, a total of 8,582 references were identified for screening at the title and abstract level. Following initial screening, 319 references were selected for full-text review. The percent agreement among reviewers for title and abstract screening was 85.1%. At the full-text stage, all 319 articles were reviewed in detail, resulting in 25 studies meeting the eligibility criteria. The percent agreement for full-text screening remained high at 81.1%. The primary reviewer monitored the percent agreement throughout both screening stages to ensure consistency and alignment among the review team. During the full-text screening process, reviewers documented reasons for exclusion for each study. A total of 294 articles were excluded.

**Figure 2:**
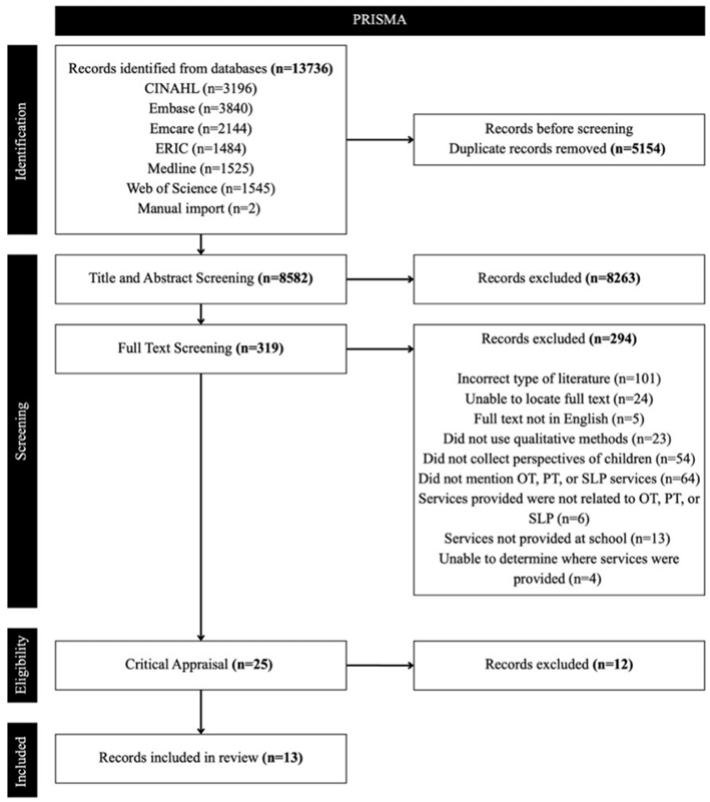
PRISMA chart of review process.

Following the screening phase, all 25 eligible studies were critically appraised using the modified JBI Critical Appraisal Checklist (58). Thirteen studies met the inclusion criteria during critical appraisal. Studies were included if they met the initial screening questions and at least six of the seven additional criteria. Ten studies met all seven methodological criteria, while three met six of the seven. Six studies were excluded for failing to meet the screening criteria, and an additional six were excluded for not meeting the methodological quality threshold. See Figure 2 for the PRISMA diagram of the study selection process.

### 3.1 Characteristics of included studies

The 13 studies included were conducted across multiple countries and continents, including the United States (*n* = 3), the United Kingdom (*n* = 2), England (*n* = 2), Australia (*n* = 1), Belgium (*n* = 1), Ireland (*n* = 1), Israel (*n* = 1), Portugal (*n* = 1), and South Africa (*n* = 1). Publication years ranged from 2009 to 2024.

A spectrum of qualitative methodologies was used across the studies: phenomenology or descriptive phenomenology (*n* = 4), grounded theory (*n* = 3), appreciative inquiry (*n* = 1), descriptive qualitative (*n* = 1), participant-focused (*n* = 1), and participatory arts-based methods (*n* = 1). Two studies did not explicitly report their methodological approach.

Participants in the included studies were children and youth from school entry to school exit, attending either mainstream or specialized schools, and with a range of conditions and disabilities. The most frequently reported condition was stuttering (*n* = 4). Other conditions included cerebral palsy (*n* = 2), chronic idiopathic neck pain (*n* = 1), cleft lip and/or palate (*n* = 1), developmental language disorder (*n* = 1), paraplegia (*n* = 1), severe learning disabilities (*n* = 1), and speech, language, and communication needs (*n* = 2). Four studies included adult participants who were reflecting on their past school experiences. The remaining studies involved children and youth aged 5–18 years. All studies focused on collecting first-person perspectives, as outlined in the eligibility criteria.

Most studies focused on SLP services (*n* = 8), while five focused on PT services. No studies focusing exclusively on OT services met the inclusion criteria. See Table 4.

**Table 4:** Relevant characteristics of selected studies for data extraction and synthesis.

**Study methods**				**Participant characteristics**			**Setting**	
**Authors**	**Phenomenon of interest**	**Methodology**	**Method of data collection**	**Data analysis approach**	**Participant age and gender**	**Disability**	**SBRS of interest**	**Location of study**
1. Alighieri et al. (59)	To investigate the perceptions, emotions and expectations of Flemish-speaking Dutch children with a cleft palate with regard to the speech therapy they receive	Participatory, art based	Child-friendly semi-structured interviews. Play and puppets technique' and ‘draw–write and photo-elicitation technique	Inductive thematic analysis	3 males, 3 females 5–12	Cleft lip/palate	Speech language pathology	Belgium
2. Allen et al. (60)	To provide an under-represented group with the opportunity to express attitudes and opinions toward their physical education (PE) lessons	Participant-focused	Task-based interviewing approach	Thematic analysis	8 male, 2 female 8–17	Severe learning disabilities	Physiotherapy	England
3. Aviram et al. (61)	Identifying the factors impacting physical activity (PA) among adolescents and young adults with cerebral palsy (CP)	Grounded theory	Focus groups	Grounded theory	11 male, 11 female 13–23	Cerebral Palsy	Physiotherapy	Israel
4. Cleary et al. (62)	To explore the perceived effects of an aerobic exercise program delivered in specialist schools for young people with cerebral palsy with high support needs	Descriptive phenomenology	In-depth interviews	Thematic analysis	5 male, 3 female 10–18	Cerebral Palsy	Physiotherapy	Australia
5. Cobb et al. (63)	To explore the ways in which adolescent students who stutter perceive their school experiences	Phenomenology	Semi-structured interviews	Not reported	4 male, 3 female 12–17	Stuttering	Speech language pathology	United States
6. Daniels et al. (64)	To explore the K-12 school experiences of adults who stutter	Phenomenology	Semi-structured interviews and focus groups	Not reported	Interviews: 8 male, 3 female 29–69 Focus group 1: 2 male, 4 female 30–58 Focus group 2: 3 male, 1 female 21–34	Stuttering	Speech language pathology	United States
7. Gallagher et al. (65)	To engage key stakeholders (SLTs, teachers, parents and children with DLD) in the co-design of their ideal speech and language therapy service and support in school	Appreciative inquiry	Semi-structured interviews using draw and tell techniques	Thematic analysis	5 male, 2 female 10–13	Developmental Language Disorder	Speech language pathology	Ireland
8. Gerlach-Houck et al. (66)	To explore experiences with concealing stuttering in children and young people who stutter based on recollections from adults	Not reported	Semi-structured interviews	Reflexive thematic analysis	16 male, 14 female 19–75	Stuttering	Speech language pathology	United Kingdom
9. Kerrigan and Brundage (67)	To explore the personal testimonies of children and adolescents who stutter	Phenomenology	Semi-structured interviews	Thematic analysis	9 male, 9 female 8–17	Stuttering	Speech language pathology	United States
10. Markham et al. (68)	To provide a qualitative, child-centerd, description of the quality of life experiences of children and young people with speech language and communication needs.	Grounded Theory	Focus groups	Grounded theory and framework analysis	22 male 7 female 6–18	Speech, language and communication needs	Speech language pathology	United Kingdom
11. Merrick and Roulstone (69)	To explore experiences of communication and of speech-language pathology from the perspectives of children with speech, language, and communication needs (SLCN)	Exploratory, Grounded Theory	Unstructured interviews	Grounded theory	7 male, 4 female 7–10	Speech, language, and communication needs	Speech language pathology	England
12. Neto et al. (70)	To explore the views of adolescents with chronic idiopathic neck pain toward an intervention consisting of pain neuroscience education and exercise administered in the school setting	Not reported	Focus groups	Content Analysis	9 male, 12 female Mean age: 17.43	Chronic idiopathic neck pain	Physiotherapy	Portugal
13. Rauter and Mathye (71)	To explore the perspectives of current and previous learners with paraplegia on peer support to prevent pressure ulcers in a special school	Descriptive qualitative	Semi-structured interviews and focus group	Inductive thematic analysis	5 male, 7 female 16–30	Paraplegia	Physiotherapy	South Africa

### 3.2 Data synthesis

Across the 13 included studies, a total of 53 findings were extracted. One finding was judged by the reviewers as unsupported and was excluded from further synthesis as per the JBI guidelines. The remaining 52 findings were assessed as credible or unequivocal and included in the final synthesis.

Based on similarities in meaning, ideas, and concepts, the 52 findings were organized into 14 categories. Twenty findings were assigned to more than one category due to including more than one idea or concept. For example, the finding titled “The leadership of the support group: Physiotherapists' role regarding peer support” (71) was categorized into two categories because it encompassed two key themes: that therapists should act as advocates and therapists should be knowledgeable. Therefore, this finding was included in both Category 12: Therapists Should Be Advocates for Children and Category 13: The Importance of Knowledgeable, Supportive, and Empathetic Therapists. Two findings remained uncategorized because they did not align with any other category, and per guidance in the JBI Manual for Evidence Synthesis (48), these findings were not synthesized further.

Finally, one category comprising two findings was not included in any of the synthesized findings as it was dissimilar from all other categories. Specifically, this category addressed loss of access to services after aging out of school or certain programs. No other categories touched on reduced access and availability of service; therefore, it was not categorized into a synthesized finding. All categories and their corresponding findings are presented in Appendix D.

**Figure 3:**
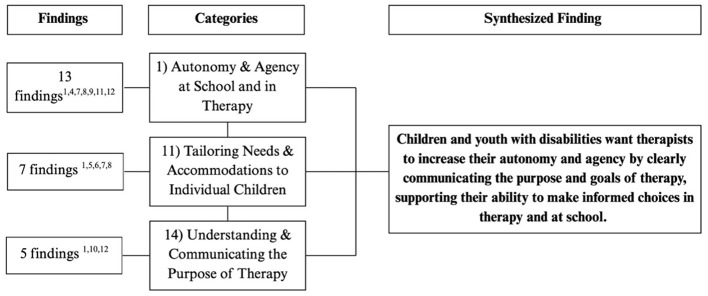
Flowchart outlining the categories and number of findings contributing to each category for final synthesized finding #1. The superscript numbers under “Findings” corresponds to the study numbers in Table 4.

**Figure 4:**
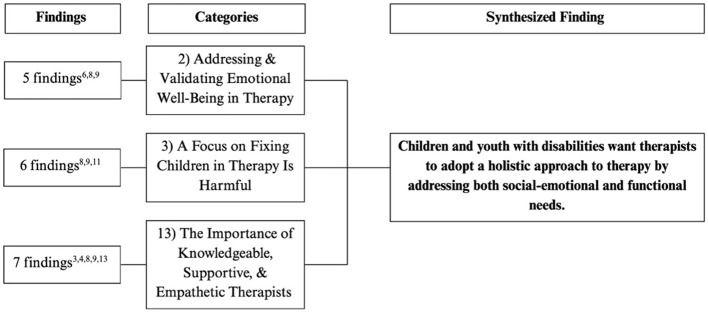
Flowchart outlining the categories and number of findings contributing to each category for final synthesized finding #2. The superscript numbers under “Findings” corresponds to the study numbers in Table 4.

**Figure 5:**
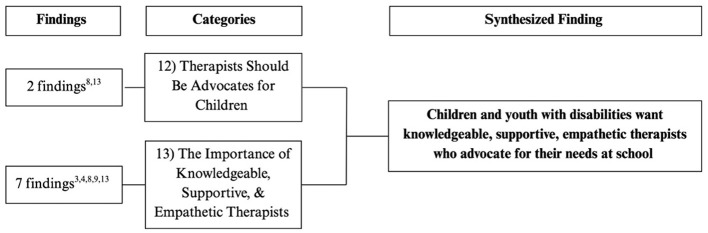
Flowchart outlining the categories and number of findings contributing to each category for final synthesized finding #3. The superscript numbers under “Findings” corresponds to the study numbers in Table 4.

**Figure 6:**
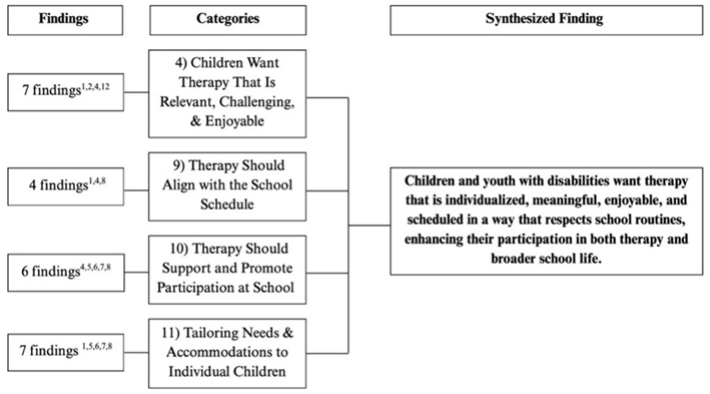
Flowchart outlining the categories and number of findings contributing to each category for final synthesized finding #4. The superscript numbers under “Findings” corresponds to the study numbers in Table 4.

**Figure 7:**
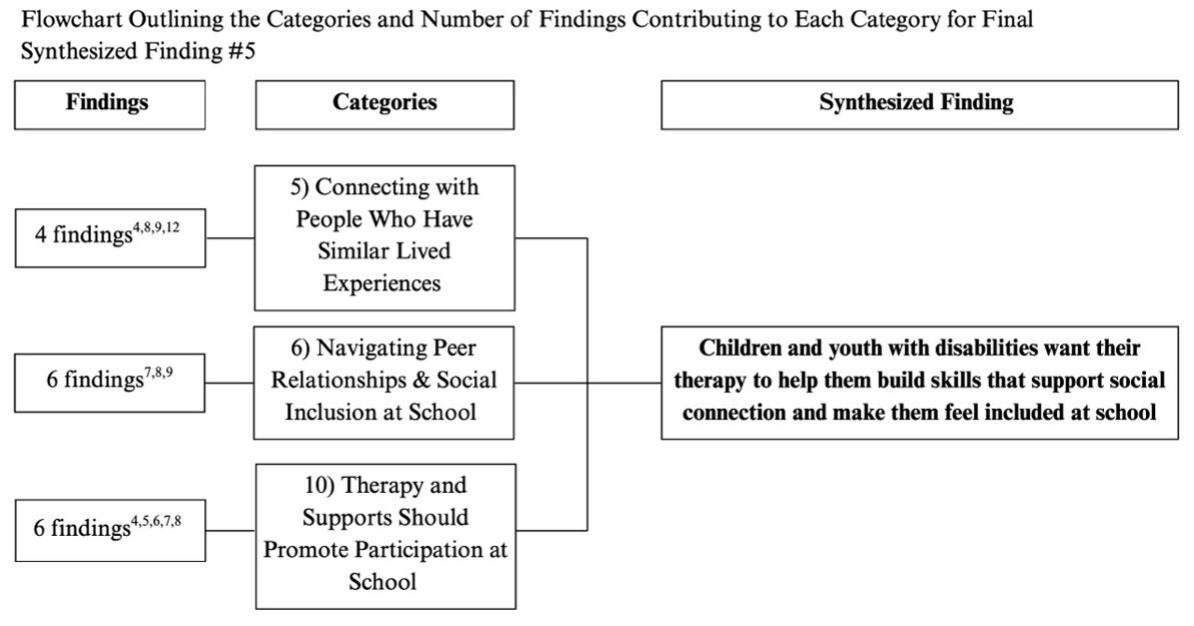
Flowchart outlining the categories and number of findings contributing to each category for final synthesized finding #5. The superscript numbers under “Findings” corresponds to the study numbers in Table 4.

**Figure 8:**
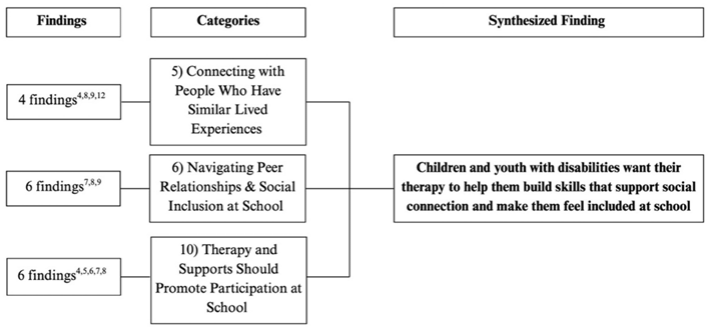
Flowchart outlining the categories and number of findings contributing to each category for final synthesized finding #6. The superscript numbers under “Findings” corresponds to the study numbers in Table 4.

The remaining 13 categories were grouped into six synthesized findings, in accordance with the JBI Manual for Evidence Synthesis (45) and are reported below. Figures 3–8 show the number of findings in each category and the categories that were grouped together into each synthesized statement.

#### 3.2.1 Synthesized finding 1: children and youth with disabilities want therapists to increase their autonomy and agency by clearly communicating the purpose and goals of therapy and supporting their ability to make informed choices in therapy and at school

Children emphasized the importance of being in control of their therapeutic experiences (Category 1) and accommodations at school (Category 11). When therapists effectively communicated the purpose of therapy (Category 14) and involved children in decision-making, it enhanced their sense of autonomy and agency (Category 1). Children who felt informed and supported were more engaged, made empowered choices, and navigated both therapy and school life with increased confidence (Category 1, 14). In contrast, when therapists did not support autonomy and agency, children experienced fear, anxiety, and shame in therapy, at school, and surrounding their disability and participation (Category 1, 11). This also depended on flexible accommodation tailored to their changing needs (Category 11).

#### 3.2.2 Synthesized finding 2: children and youth with disabilities want therapists to adopt a holistic approach to therapy by addressing both social-emotional and functional needs

Children expressed the desire for therapy to support not only the medical aspects of their condition but also their social and emotional well-being (Category 2). They wanted a safe space to openly discuss their emotions and feel understood by therapists who acknowledged the challenges of living with a disability (Category 2). When therapists focused solely on “fixing” their condition without validating their emotional experiences, children felt invalidated, misunderstood, and frustrated (Category 3). These concerns were particularly evident among children receiving SLP services. Conversely, therapists who were knowledgeable, empathetic, supportive, and approachable made children feel seen and heard, which helped them better manage the emotional challenges related to their condition (Category 13). Children emphasized that therapists should also encourage acceptance of their disability as part of their identity rather than promoting the idea that it must be fixed or changed (Category 2, 3).

#### 3.2.3 Synthesized finding 3: children and youth with disabilities want knowledgeable, supportive, empathetic therapists who advocate for their needs at school

Children appreciated therapists who were well-informed about their disability and could educate others within the school environment (Category 13). These therapists created a safe and encouraging space where children felt comfortable sharing their challenges and experiences (Category 13). Additionally, children valued therapists who were strong advocates by helping ensure their needs were understood and supported by teachers, peers, and other school staff. Advocacy involves raising awareness about the child's condition and fostering greater understanding within the school community (Category 12).

#### 3.2.4 Synthesized finding 4: children and youth with disabilities want therapy that is individualized, meaningful, enjoyable, and scheduled in a way that respects school routines, enhancing their participation in both therapy and broader school life

When children received therapy that was personalized, engaging, and relevant to their interests (Category 4), without interrupting valued school activities (Category 9), they were more motivated to participate in both therapy and the broader school environment (Category 10). Children emphasized the importance of therapy being well-timed within the school day to avoid conflicts with learning, social opportunities, or preferred classroom activities (Category 9). When therapy aligned with their needs, interests, and routines (Category 4, 9, 11), it supported children's ability to participate fully at school (Category 10). Children also wanted academic accommodations to be implemented flexibly and reflect that their support needs may change from one day to the next or from one class to the next (Category 11).

#### 3.2.5 Synthesized finding 5: children and youth with disabilities want their therapy to help them build skills supporting social connection and making them feel included at school

Children emphasized the importance of being able to connect socially with peers and others, including those with similar lived experiences, therapists, and educators (Category 5, 6). They wanted therapy to help them understand social expectations and build the skills needed to interact confidently with others and feel included at school (Category 6). Building relationships with peers who shared similar experiences was especially meaningful, as it provided a sense of understanding and connection that others could not offer (Category 5). Children also valued therapy and school staff that supported their inclusion in everyday social situations, helping them learn how to initiate, respond to, and sustain interactions with peers, and how to advocate for themselves in challenging social moments (Category 6). Overall, therapy that promoted participation in school life helped children feel more engaged and less scared to join activities and interact with peers (Category 10).

#### 3.2.6 Synthesized finding 6: children and youth with disabilities want to set goals and develop new skills in therapy

Children viewed therapy as a supportive space to set meaningful goals and to learn, practice, and improve new skills (Category 7). They valued therapists who communicated clearly the purpose of therapy, helping them understand why they were there and how to engage in goal setting and skill development (Category 14). A non-judgmental environment that encouraged effort, allowed room for mistakes, and provided consistent opportunities for skill-building made therapy meaningful and motivating (Category 7).

## 4 Discussion

This meta-aggregative review aimed to identify the wants and needs of children and youth with disabilities regarding their SBRS. By synthesizing findings from 13 primary high-quality studies, this review generated six synthesized statements that offer evidence-based recommendations intended to guide policymakers and school-based practitioners. Specifically, the findings underscore that children and youth with disabilities wish to participate meaningfully and fully in all aspects of school life, emphasizing the importance of autonomy not only in academic settings but also within therapeutic interactions. They also expressed a strong desire to feel included in the school community and to form meaningful connections with peers and adults. Furthermore, participants highlighted the need for socially informed and holistic care that recognizes their broader life contexts. A recurring theme was the importance of having therapists who are supportive, respectful, and responsive to their individual needs. Finally, children and youth with disabilities value opportunities to engage in goal setting and skill development activities that are personally meaningful and directly relevant to their everyday lives.

### 4.1 Implications for SBRS practice and policy

This section outlines key implications for SBRS practice and policy, with emphasis on the importance of supporting children's autonomy in therapy and at school, providing holistic and socially informed approaches to therapy, and aligning SBRS delivery with inclusive values. We build on the implications for practice to highlight what is important for policymakers.

#### 4.1.1 The importance of autonomy-supportive therapy

Self-Determination Theory (SDT) is a well-established framework for understanding human motivation and personality (72). It has been widely applied in several fields, including education and healthcare, to explain human behavior and motivation (72, 73). SDT proposes that humans have an inherent drive toward self-actualization, which depends on the fulfillment of three basic psychological needs: autonomy, competence, and relatedness (72). These needs are essential for fostering motivation, development, and overall well-being (72, 74). Ryan and Deci define autonomy as “the need to self-regulate one's experiences and actions” [(75), p. 10], emphasizing that autonomous behaviors are self-endorsed and align with personal interests and values (75). Our findings can be interpreted through this lens, as children and youth with disabilities expressed a strong desire to develop autonomy with the support of their therapists.

Considering the central role of autonomy in well-being, it is notable that adults often share an underlying assumption that children lack autonomy due to perceived cognitive or emotional immaturity (76, 77). However, the argument that children lack autonomy has been challenged as researchers increasingly recognize that children can think rationally, demonstrate willpower, and be active shapers of their own lives (3, 77, 78). Rather than denying autonomy, researchers, therapists, and caregivers must foster it through autonomy-supportive practices that help build capacity over time (77, 79, 80). When provided with appropriate support, children can make meaningful choices and contribute to decisions that affect them (77, 79, 80).

Children and youth with disabilities often face challenges in achieving autonomy (81). Yet, our findings suggest that children and youth with disabilities value being informed and involved in decisions about their therapy and school experiences [e.g., (59, 65–70)]. Interestingly, Antoniadou et al. reported in their scoping review that autonomy was fostered when rehabilitation professionals provided clear and age-appropriate explanations of therapy goals, set explicit expectations, and aligned therapy activities with the child's interests (82). Strategies shared in the scoping review included communicating schedules through visual and verbal means and seeking children's input on therapy agendas and activities (82). Rehabilitation professionals also could use collaborative goal-setting tools, including the Perceived Efficacy and Goal Setting System (83) and the Canadian Occupational Performance Measure (84), to support meaningful participation in therapy goal setting. Additionally, tools like “Skills for Growing Up” have been found to enhance communication between youth, parents and professionals, and empower youth with disabilities to take ownership of therapy decisions (81).

Similar autonomy-supportive practices have been reflected and used in education settings (85). For example, a systematic review by Yang et al. (85) found that teachers who adopted autonomy-supportive practices (e.g., collaborative rule-setting, setting clear goals and expectations, eliciting students' perspectives, providing rationales, and supporting emotions) maximized student engagement and fostered autonomy-supportive learning environments (85). These practices closely align with what the children in our review described as supportive: being clearly informed about therapy goals and procedures (59, 68, 70), having their interests respected (59, 65–67), and being actively involved in shaping their therapy experience (59, 65–67, 69).

Together, these findings highlight the value of autonomy-supportive practices in SBRS. Therapists may support autonomy by offering meaningful choices, respecting children's preferences, and involving them in therapy-related decision-making and goal setting.

**Figure 9:**
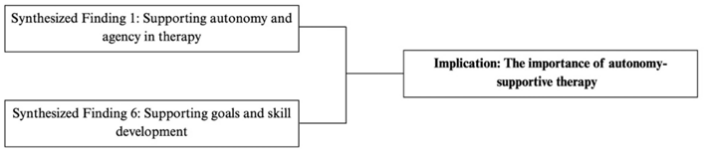
Flowchart outlining synthesized findings that contributed to practice implication: autonomy-support.

Figure 9 outlines the synthesized statements that informed the autonomy-supportive practice implications.

#### 4.1.2 Adopt a holistic, social informed approach

Building autonomy in therapy requires not only the right environment and therapist support but also alignment with the models and frameworks that inform service delivery. The medical model of disability, which frames disability as an individual deficit requiring correction (86), continues to shape how rehabilitation services are designed and delivered despite recognized limitations (86). Notably, this model directly contradicts what children and youth with disabilities want and value: for therapy experiences to be empowering and validating rather than corrective (64, 66, 67).

Findings from this review indicated that children and youth had a strong desire for SBRS to recognize their identities as individuals with disabilities, including their emotional, social, and functional needs (64, 66, 67). They described medicalized approaches focused solely on “fixing” impairments as invalidating, frustrating, and disconnected from their lived experiences, sometimes creating feelings of shame surrounding their disability (64, 66, 67). Instead, they preferred therapy and school support that were individualized, meaningful, and responsive to their interests, emotions, and routines (59, 62, 64, 66, 67).

The social model of disability offers an alternative framework for understanding disability and informing approaches to rehabilitation (86, 87). The social model of disability frames disability as the result of structural, social, and environmental barriers that limit participation rather than locating the problem within individuals (86, 88). This model affirms disability as part of human diversity and emphasizes emotional well-being, inclusion, and autonomy (86–89). Studies included in our review reported that children articulated these same priorities, expressing a desire for therapy that went beyond functional or health goals, to also support their emotional and social well-being (64, 66, 67). They wanted their disabilities, feelings, and experiences to be acknowledged by therapists (64, 66, 67), and sought support in developing the skills and strategies needed to build social connections at school (65–67).

These findings suggest that school-based therapists should adopt a holistic and socially informed approach that centers children's emotional experiences, values their perspectives, and affirms disability as an aspect of identity. Such approaches require shifting away from deficit-oriented practices toward models that prioritize emotional support, social belonging, personal agency, and inclusive participation.

**Figure 10:**
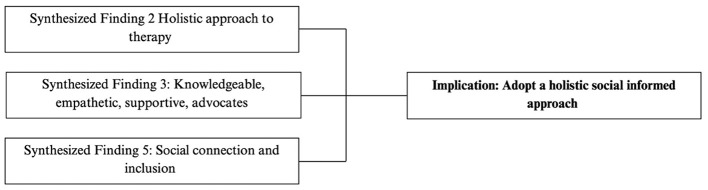
Flowchart outlining synthesized findings that contributed to practice implication: holistic approach.

Figure 10 outlines the synthesized statements that informed the holistic, socially informed practice implications.

#### 4.1.3 Align SBRS delivery with inclusive values

Globally, there is an increasing shift from segregated schooling toward inclusive education, reflected in national and international policies that aim to ensure all children and youth have access to quality education within their school environments (26–28). Inclusive education is grounded in values of equity, participation, and social justice (26, 27). Children and youth with disabilities in inclusive settings demonstrate positive outcomes academically and socially (90). For example, Katz et al. (91) found that the use of inclusive classroom strategies led to improved academic performance across children with and without disabilities. Importantly, inclusive environments also foster a sense of community and are associated with greater peer acceptance, improved social relationships, fewer instances of teasing, and reduced feelings of loneliness (90, 92).

Our findings reinforce that children and youth receiving SBRS want services to reflect inclusive values and practices. Children and youth emphasized that therapy felt most meaningful when it supported peer interaction (65–67), encouraged social participation (65, 66), provided appropriate accommodations (63, 66), and enhanced engagement and participation in school life and therapy (59, 62, 65). They valued services that were embedded in the everyday rhythm of school, supporting their sense of belonging and enabling them to participate as active members of their school communities (59, 62, 66).

As school systems become increasingly inclusive, SBRS must evolve away from solely using pull-out models of service that remove children from the classroom for remediation toward classroom-based, collaborative approaches that support children's functioning and participation (24, 93, 94). School-based therapists play a key role in fostering inclusive environments by adapting interventions to fit classroom contexts, supporting social and academic participation, and advocating for educational accommodations and systemic supports (24, 94). Rather than functioning in isolation, therapists must collaborate with educators, families, and school staff to design supports that are responsive to children's needs while also promoting inclusive participation (24, 93, 97). Tiered service models that offer a continuum of support from universal to individualized exemplify this shift by providing more integrated, inclusive, and flexible supports (24, 93, 98).

As children and youth voice their wants and needs for inclusion at school, it is crucial that SBRS service delivery and practice align with these values. Tiered, collaborative models of service delivery are designed and delivered to respect these inclusive values (24, 93, 94); however, more research is needed from the perspectives of children to determine if these services are working optimally to support children and youth with disabilities at school.

**Figure 11:**
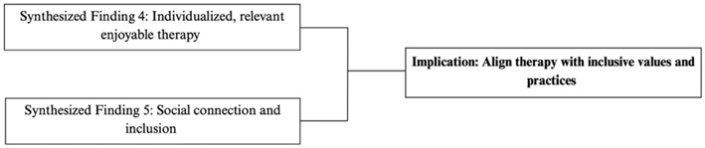
Flowchart outlining synthesized findings that contributed to practice implication: inclusive values.

Figure 11 outlines the synthesized statements that informed the inclusive practice implications.

#### 4.1.4 Policy implications

Building on the practice implications outlined above, the findings of this review also have implications for policies that govern SBRS. For example, policymakers need to consider whether SBRS models and guiding policies enable therapists to deliver child-centered, autonomy-supportive, holistic, and inclusive services within schools. Many existing policies remain rooted in deficit- and impairment-focused frameworks (22, 95, 96). In contrast, our findings suggest a need for policies grounded in socially informed and affirmative approaches to disability that recognize children's social, emotional, and functional needs and support inclusive service delivery models embedded in everyday school routines (24, 96–98). To enable SBRS therapists to make meaningful change, policies also must allocate adequate time and resources for professional development and mentorship to support shifts toward autonomy-supportive and inclusive practices within school systems (24, 97, 99–102).

Finally, policies should support the meaningful involvement of children and youth with disabilities in decision-making processes related to service design, delivery, evaluation, and research. Embedding children's voices in policy development is essential to uphold their rights and ensure policies reflect the priorities and lived experiences of children and youth (3, 103, 104).

#### 4.1.5 Future training needs for therapists

To support autonomy, holistic care and inclusive service delivery, school-based therapists require targeted and ongoing professional development. Training should focus on autonomy-supportive practices (e.g., collaborative goal setting), and strategies for meaningfully involving children in their therapy and school experiences (75, 81). In addition, therapists require training in socially informed and affirmative approaches to disability that emphasize children's social, emotional, and functional needs, and move beyond deficit-oriented frameworks (97, 100–103). Therapists also need guidance, mentorship, and time to effectively implement inclusive service delivery approaches, such as tiered models of support, which often require shifts in traditional service delivery practices (24, 98). These training needs highlight the importance of policies and systems that provide professional development opportunities and training to support the delivery of autonomy-supportive, holistic, and inclusive school-based services.

### 4.2 Strengths and limitations

One of the key strengths of this meta-aggregative review was our iKT approach, which actively involved youth with disabilities throughout the research process. Review team members with lived experience contributed from protocol development through to the final synthesis, including verifying review findings, categories, and synthesized statements to support alignment with lived experiences. It is important to note that although youth engagement throughout the research process was considered a strength of the review, it also required additional time and resources to teach youth new research skills, especially as their familiarity with conducting research reviews varied.

Regular team meetings facilitated peer debriefing and ongoing discussions about the research process. The primary reviewer also met independently and regularly with an external reviewer, who is an experienced qualitative researcher specializing in SBRS and service delivery, to discuss the research process, decisions, and to verify interpretations. This encouraged a reflexive process, and the primary researcher kept reflexive notes documenting key decisions and methodological adjustments. This process increased confidence that our synthesis incorporated multiple valuable perspectives rather than relying solely on the viewpoint of the primary reviewer.

Our review also had some limitations. First, despite efforts to develop a comprehensive search strategy, some relevant literature may not have been identified. The broad scope of our review question made it challenging to create a targeted yet comprehensive search strategy, potentially affecting the retrieval of all relevant studies. Because our research focused on lived experiences and perspectives, we encountered variability in how these concepts were described across studies. There is no universal terminology used to represent lived experience or first-person perspectives in research, meaning some studies may have captured these elements without explicitly stating so. To address this, we incorporated search terms related to qualitative research to identify studies that may have explored these concepts indirectly. This decision contributed to a broader, more comprehensive search. However, despite these efforts, key information needed to determine study eligibility was often located only in the full text or methods section rather than the abstract, which may have led to the omission of some relevant studies during screening.

A second limitation concerns the critical appraisal process, which may have excluded studies containing relevant information. The JBI methodology includes this step because a key aim of meta-aggregation is to develop recommendations for policy and practice, necessitating the inclusion of studies with high methodological quality (45, 105). Nevertheless, it is possible that some studies with relevant findings were excluded at this stage, potentially limiting the breadth of our final synthesis. Although there is ongoing debate about whether studies should be included regardless of methodological quality (106), adherence to JBI guidelines made this a crucial component of our review.

Finally, it is possible that publication bias may have limited the literature included in this review. In qualitative research, publication bias, also referred to as dissemination bias, can influence the body of available evidence (107). Dissemination bias occurs when studies are less likely to be published because their results are considered less noteworthy, contradict prevailing beliefs or values, are unpopular, or do not align with current or anticipated research agendas (107, 108). Future research is needed to examine how dissemination bias specifically affects qualitative evidence syntheses and the impact it has on the use of qualitative findings to inform policy and practice (107).

Despite these limitations, the rigorous methodology and inclusion of high-quality qualitative studies strengthen confidence in the findings. While potentially missed studies may have contributed additional findings and perspectives, the aggregated findings represent a credible and transparent synthesis of the best available qualitative evidence identified, supporting transferability to similar populations and contexts.

### 4.3 Future research

As we were unable to locate any relevant studies representing school-based OT services, one area for future exploration would be to engage children and youth receiving OT services to better understand their specific wants and needs. In addition, the literature disproportionately represented older children and youth, with limited inclusion of younger children (60–63, 65, 67, 70, 71). This overrepresentation highlights the importance of future research that captures more diverse perspectives across ages, as well from children and youth with a broader range of disabilities, abilities, and service needs. Child-centered and participatory qualitative methods are particularly well suited for engaging children and youth in research. Methods such as draw-and-tell techniques, task-based and play-based approaches, and visual methods including photovoice or photo elicitation allow children to express their perspectives in ways that extend beyond verbal communication alone (41, 42, 109, 110). These child-led approaches can enhance accessibility, reduce power imbalances, and support more authentic representation of children's lived experiences in research (41, 42, 109, 110).

Future research should also address the predominance of Western and Anglo-centric perspectives in the existing literature. In this review, the majority of included studies were conducted in the United States (63, 64, 67) and the United Kingdom (8, 60, 65, 66, 69), underscoring the need to incorporate more international perspectives by engaging children and youth from a broader range of countries, cultures, geographic regions, and socio-economic and political contexts. Similarly, the inclusive education literature remains largely concentrated in developed, Western, and Anglo-centric countries, where research, policy development, and documentation of inclusive practices have been more extensively established (111). This concentration limits understanding of how inclusive education and SBRS are implemented and experienced in developing countries and culturally diverse settings. Expanding research beyond Western and Anglo-centric contexts is therefore essential to support the development of more globally relevant, equitable, and culturally responsive evidence to inform policy and practice.

### 4.4 Key messages for policy makers

- Shift SBRS policies from deficit-focused frameworks to socially informed, affirmative approaches that recognize children and youth with disabilities as active participants in school life.
- Ground SBRS policies in inclusive values by supporting services that promote participation, belonging, and integration within everyday school routines.
- Allocate sufficient time and resources for professional development and mentorship to support therapists in implementing autonomy-supportive, holistic, and inclusive practices.
- Meaningfully involve children and youth with disabilities in policy development, service design, and evaluation to ensure policies reflect their priorities and lived experiences.

## 5 Conclusion

The results of this review highlighted that children and youth with disabilities have multiple wants for their SBRS based on their lived experiences. They want therapy to support their participation in meaningful ways by being relevant, individualized, appropriately challenging, and respectful of the school schedule. Participation, as described by children, goes beyond functional improvement; it includes opportunities to connect socially with peers and adults, fostering a greater sense of inclusion within the school environment. Children also expressed a desire for therapy to be a space where they can set meaningful goals and build skills that enable them to engage more fully in school life. They wanted to strengthen their autonomy and agency by being equipped with the knowledge and tools to navigate school successfully and be an active participant in their therapy decisions. Lastly, they called for a more holistic approach to therapy, which supports both their medical and social and emotional needs. The findings should be considered in the delivery and policies of SBRS.
