# Supplementary material for: Understanding school-based rehabilitation services through the lived experiences of children and youth with disabilities: a meta-aggregative review
Source: Front Public Health. 2026 Feb 27;14:1745224. doi: 10.3389/fpubh.2026.1745224 (PMC12982189; doi:10.3389/fpubh.2026.1745224)
Supplement: Supplementary file 1 [file Table_1.DOCX]

## **Appendix A**

*Search Strategies*

*CINAHL Search Strategy*

| CINAHL  Run on October 17^th^, 2024 | Population  (Children & Youth) | Interest  (Perspectives & Qualitative Terms) | Interest (Rehabilitation) | Context  (School) |
| --- | --- | --- | --- | --- |
| Keywords | "child*"  "teen*”  "adolescen*"  "pre-teen*"  "preteen*"  "youth*" | "attitude*”  "experience*"  "perspective*"  "perception*"  "response*"  "respond*"  "opinion*"  "belief*"  "view*"  "lived experience*” OR "lived-experience*"  “life experience*”  phenomenolog*  "qualitative"  "ethnograph*"  “grounded theor*”  "hermeneutic*"  "heuristic*"  "semiotic*"  "narrative*"  "action research"  “content analys?s"  "discourse analys?s"  "group discussion*"  "focus group*"  "anthropology"  "anthropological"  "ethnolog*"  "interview*"  "photo elicitation" OR "photoelicitation"OR "photo-elicitation"  "autophotograph*"  "visual ethnograph*"  "participatory photograph*"  "ethnomethodolog*"  "arts based method*"  "arts based research" | "occupational therap*"  “physiotherap*”  "physical therap*"  "speech language patholog*"  "speech language therap*"  "language therap*"  "speech therap*" | "school*"  "school base*"  "high school*"  "secondary school*"  "primary school*"  "middle school*"  "elementary school*"  "junior high*"  "kindergarten*" |
| Subject Headings | exp child/  exp adolescence/ | (MH "Life Experiences+")  (MH "Perception+")  (MH "Attitude+")    (MH "Qualitative Studies+")  (MH "Anthropology+")  (MH "Content Analysis")  (MH "Discourse Analysis")  (MH "Discussion")  (MH "Focus Groups")  (MH "Ethnographic Research")  (MH "Phenomenology")  (MM "Interviews")  (MH "Narratives+") | (MH "Occupational Therapy+")  (MH "Occupational Therapists+")  (MH "Speech-Language Pathology")  (MH "Rehabilitation, Speech and Language+")  (MH "Physical Therapy+")  (MH "Physical Therapists+") | (MH "Schools, Elementary")  (MH "Schools, Middle")  (MH "Schools, Secondary")  (MH "Schools, Special")  (MH "School Health Services+") |

*Embase & Emcare Search Strategy*

| EMBASE & EMCARE Search  Run on October 17^th^, 2024 | Population  (Children & Youth) | Interest  (Perspectives & Qualitative Terms) | Interest (Rehabilitation) | Context  (School) |
| --- | --- | --- | --- | --- |
| Keywords | child*.mp.  youth* .mp.  teen*.mp.  adolescen*.mp.  pre-teen*.mp. Or preteen* | attitude* .mp.  perspective* .mp.  perception* .mp.  experience* .mp.  response*.mp.  responsd*.mp  opinion*.mp.  belief*.mp.  view*.mp.  phenomenolog*.mp.  qualitative*.mp.  ethnograph*.mp.  grounded theor*.mp.  hermeneutic*.mp.  heuristic*.mp.  semiotic*.mp.  narrative*.mp.  lived experience* .mp.  life experience*.mp.  action research.mp.  content analys?s.mp.  discourse analys?s.mp.  ethnomethodolog*.mp.  participatory photograph*".mp.  visual ethnograph*.mp.  autophotograph*.mp. OR auto photograph*.mp.  photoelicitation.mp. OR photo elicitation".mp.  photovoice.mp OR photo voice*.mp.  interview*.mp.  ethnolog*.mp.  anthropological.mp.  anthropology.mp.  focus group*.mp.  group discussion*.mp.  arts based method*.mp. | occupational therap*.mp.  physiotherap*.mp.  physical therap*.mp.  speech language patholog*.mp.  speech language therap*.mp.  language therap*.mp.  speech therap*.mp. | school*.mp.  school base* .mp.  high school*.mp.  elementary school*.mp.  secondary school*.mp.  middle school*.mp.  junior high*.mp.  primary school*.mp.  kindergarten*.mp. |
| Subject Headings | exp child/  exp adolescence/ | exp attitude/  exp experience/  exp personal experience/  exp interview/  exp anthropology/  exp discourse analysis/  exp content analysis/  exp participatory action research/ or exp action research/  exp ethnology/  exp heuristics/  exp hermeneutics/  exp grounded theory/  exp phenomenology/  exp ethnography/  exp qualitative research/ | exp speech therapy/ or speech rehabilitation/  exp speech language pathologist/  exp occupational therapy/  exp occupational therapist/  exp physiotherapist/  exp physiotherapy/ | exp school health service/  high school/ or kindergarten/ or middle school/ or primary school/ |

*Eric Search Strategy*

| ERIC Search  Run on October 18^th^, 2024 | Population  (Children & Youth) | Interest  (Perspectives & Qualitative Terms) | Interest (Rehabilitation) | Context  (School) |
| --- | --- | --- | --- | --- |
| Keywords | teen*  adolescen*  pre-teen*  preteen*  child*  youth* | attitude*  perspective*  perception*  experience*  response*  respond*  opinion*  view*  belief*  phenomenolog*  qualitative*  ethnograph*  grounded theor*  hermeneutic*  heuristic*  semiotic*  narrative*  lived experience*  lived-experience*  life experience*  action research*  content analys?s*  discourse analys?s*  ethnomethodolog*  participatory photograph*  visual ethnograph*  autophotograph*  auto photograph*  photoelicitation*  photo elicitation*  photovoice*  photo voice*  interview*  ethnolog*  anthropological  anthropology  focus group*  group discussion*  arts based method* | occupational therap*  physiotherap*  physical therap*  speech language patholog*  speech language therap*  language therap*  speech therap* | school*  school base*  elementary school*  high school*  middle school*  primary school*  secondary school*  junior high*  kindergarten* |

*Medline Search Strategy*

| MEDLINE Search  Run on October 17^th^, 2024 | Population  (Children & Youth) | Interest  (Perspectives & Qualitative Terms) | Interest (Rehabilitation) | Context  (School) |
| --- | --- | --- | --- | --- |
| Keywords | child*.mp.  youth* .mp.  teen*.mp.  adolescen*.mp.  pre-teen*.mp. Or preteen* | attitude* .mp.  perspective* .mp.  perception* .mp.  experience* .mp.  response*.mp.  responsd*.mp  opinion*.mp.  belief*.mp.  view*.mp.  phenomenolog*.mp.  qualitative*.mp.  ethnograph*.mp.  grounded theor*.mp.  hermeneutic*.mp.  heuristic*.mp.  semiotic*.mp.  narrative*.mp.  lived experience* .mp.  life experience*.mp.  action research.mp.  content analys?s.mp.  discourse analys?s.mp.  ethnomethodolog*.mp.  participatory photograph*".mp.  visual ethnograph*.mp.  autophotograph*.mp. OR auto photograph*.mp.  photoelicitation.mp. OR photo elicitation".mp.  photovoice.mp OR photo voice*.mp.  interview*.mp.  ethnolog*.mp.  anthropological.mp.  anthropology.mp.  focus group*.mp.  group discussion*.mp.  arts based method*.mp. | occupational therap*.mp.  physiotherap*.mp.  physical therap*.mp.  speech language patholog*.mp.  speech language therap*.mp.  language therap*.mp.  speech therap*.mp. | school*.mp.  school base* .mp.  high school*.mp.  elementary school*.mp.  secondary school*.mp.  middle school*.mp.  junior high*.mp.  primary school*.mp.  kindergarten*.mp. |
| Subject Headings | exp child/  exp adolescence/ | exp attitude/  exp interview/  exp anthropology/  exp ethnology/  exp heuristics/  exp hermeneutics/  exp grounded theory/  exp ethnography/  exp qualitative research/ | exp speech therapy/ or speech rehabilitation/  exp speech language pathologist/  exp occupational therapy/  exp occupational therapist/  exp Physical Therapy Modalities/  exp physiotherapy/ | exp school health service/ |

*Web of Science Search Strategy*

| WOS Search  Run on October 18^th^, 2024 | Population  (Children & Youth) | Interest  (Perspectives & Qualitative Terms) | Interest (Rehabilitation) | Context  (School) |
| --- | --- | --- | --- | --- |
| Keywords | teen*  adolescen*  pre-teen*  preteen*  child*  youth* | perspective*  perception*  response*  respond*  opinion*  belief*  view*  "lived-experience*"  "life experience*"  phenomenolog*  qualitative*  ethnograph*  "grounded theor*"  hermeneutic*  heuristic*  semiotic*  narrative*  "action research*"  "content analys?s*"  "discourse analys?s*"  "group discussion*"  "focus group*"  anthropology  anthropological  ethnolog*  interview*  photoelicitation*  "photo elicitation*"  "photo-elicitation*"  autophotograph*  "auto photograph*"  "visual ethnograph*"  "participatory photograph*"  ethnomethodolog*  "arts based method*"  "arts based research*" | “occupational therap*”  physiotherap*  "physical therap*"  "speech language therap*"  "speech language patholog*"  "speech therap*"  "language therap*" | school*  "school base*"  "school-base*"  "high school*"  "secondary school*"  "primary school*"  "middle school*"  "elementary school*"  "junior high*"  kindergarten* |
