# Supplementary material for: Understanding school-based rehabilitation services through the lived experiences of children and youth with disabilities: a meta-aggregative review
Source: Front Public Health. 2026 Feb 27;14:1745224. doi: 10.3389/fpubh.2026.1745224 (PMC12982189; doi:10.3389/fpubh.2026.1745224)
Supplement: Supplementary file 2 [file Table_2.DOCX]

## **Appendix B**

*Eligibility Criteria*

1. Language

- Included: Studies published in English
- Excluded: Studies published in languages other than English

1. Methods

- Included: Qualitative and mixed methods
- Excluded: Quantitative methods

1. Research Type

- Included: Primary research conducted by the authors
- Excluded: Literature reviews or secondary analyses

1. Literature

- Included: Scholarly sources such as peer-reviewed journal articles, book chapters, dissertations, and theses
- Excluded: Non-scholarly sources such as government documents, conference proceedings, letters to the editor, tutorials, and guides

1. Population

- Included: Children and youth from school entry to school exit and individuals reflecting on their school-age experiences
- Excluded: Non-child perspectives (e.g., parents, educators, healthcare providers) and children and youth of preschool age

1. Perspectives

- Include: First-person perspectives
- Excluded: Second-hand recounts (e.g., adults speaking on behalf of children)

1. Context

- Included: Mainstream and specialized school settings (e.g., public/private, primary, elementary, middle, junior high, secondary/high schools)
- Excluded: Non-K–12 schooling environments such as preschool and post-secondary institutions and settings outside of school, such as daycares, clinics, community programs, and camps

1. Services

- Include: Services delivered by occupational therapists, physiotherapists, speech language pathologists, and their respective assistants
- Exclude: Services provided by psychologists, nurses, social workers, or dietitians
