# Supplementary material for: Understanding school-based rehabilitation services through the lived experiences of children and youth with disabilities: a meta-aggregative review
Source: Front Public Health. 2026 Feb 27;14:1745224. doi: 10.3389/fpubh.2026.1745224 (PMC12982189; doi:10.3389/fpubh.2026.1745224)
Supplement: Supplementary file 3 [file Table_3.DOCX]

## **Appendix C**

*Critical Appraisal Checklist and Guidelines*

**Screening Questions**

**1.**  **Is there congruity between the research methodology and the research question or objective?**

This question is asking if the study methodology is appropriate for addressing the authors’ research question or objective. Statement(s) about the research question and/or objective of the study can be found towards the end of the introduction of the paper. The methodology can be found by consulting the methods of the paper. Both the research question/objective and the methodology may also be captured in the abstract.

To answer this question, consider if the authors clearly stated their research question or objective, and if so, what was it? Then consider if qualitative research is the right methodology for addressing that question or objective. Qualitative research seeks to explore or interpret meanings of experiences, actions, and/or beliefs (*CASP Qualitative Checklist*, 2018). Does this align with the research question/objective?

| **Answer YES, if** | **Answer NO, if** |
| --- | --- |
| - **Authors include a clear statement about their research question/ objective** - **To be considered clear, a reader would be able to identify this text in the article** - **Note that some authors may refer to this as their research objective or question, while other authors may use the terms research goal or aim.** - **Authors’ intention is to explore a phenomenon; specifically, describing, interpreting or illuminating the meanings of experiences, actions, and/or beliefs of people** | - Authors’ research question or objective is not clearly stated - Unable to locate any text specifically stating the research question or objective - If text is present, the wording is unclear or confusing. - The question or objective has to be inferred by reading the rest of the paper. - Authors’ question or objective is not consistent with qualitative research methodology (i.e., does not seek to explore a phenomenon) - Does not seek to explore or interpret the meanings of experiences, actions, and/or beliefs of people - Authors’ question or objective is more consistent with quantitative research methodology (i.e., seeks to confirm hypotheses about phenomena, predict causal relationship, and/or describe characteristics of a population) (Mack et al., 2005) |

**2.        Are participants, and their voices, adequately represented?**

Generally, reports should provide illustrations from the data to show the basis of their conclusions and to ensure that participants are represented in the report. So, this question is asking if the findings/results adequately represent the voices of the children and youth with disability or special needs.

To answer this question, consider if original quotes from the participants are referenced throughout the results section, specifically in each theme/category (Hannes et al., 2013). Are they full direct quotes (McTavish et al., 2017), or small excerpts/partial phrases worked into the author’s interpretations? Does the author include participant pseudonyms or ID numbers with the quotes? Are the quotes representative of ALL participants?

| **Answer YES, if** | **Answer NO, if** |
| --- | --- |
| - **Original quotes from participants are referenced in each theme/category** - **The quotes are predominantly full, direct quotes** - **Note: If there are few or no full quotes, check the participant criteria to determine the age or communication abilities of participants (e.g., children or youth who are AAC users or very young children with communication difficulties who may not speak in full or complex sentences); in such cases, it would be appropriate for the participants to have partial rather than full quotes.** - **It is clear which participant each quote is from, and the quotes represent ALL participants** | - Original quotes from participants are not referenced throughout (e.g., quotes are presented for only a section of the results) - The quotes are predominantly partial excerpts/short phrases worked into the author’s interpretations (and this cannot be explained by the participants’ age or communication status) - It is not clear which participants the quotes are from, or there is not enough variation in the sources of quotes and are not representative of ALL study participants. |
| **Example of a full direct quote: Emma used her phone. “I often will take them [images] on my phone. It’s annoying because then you have to go home and write them down. It just takes more of your time, but yeah, I do that if I have missed things.” (Opie, 2018, p.655)** | Example of a partial excerpt or short phrase quote: Emma did not want to appear any different to her peers, and was reluctant to ‘go against the rules’ She did not feel she could ask teachers for permission to use her computer in class as this might have been seen as ‘having an advantage’, so she suffered in silence, although it meant relying on others. (Opie, 2018, p.654) |

**If either of these two questions are answered “no”, the study is excluded. If both questions are answered “yes”, proceed to the remaining questions.**

**3.        Is the research ethical according to currently accepted criteria, and is there evidence of ethical approval by an appropriate body?**

A statement on the ethical approval process followed should be in the report, usually found in the methods section.

To answer this question, consider if the researcher used at least **two** of the following strategies to ensure ethical issues were taken into consideration: 1) it is sufficiently clear how the research was explained to participants for the reader to assess whether ethical standards were maintained; 2) the researcher discussed ethical issues that arose in  the study, such as issues around informed consent or confidentiality or how they have handled the effects of the study on the participants during and after the study; or 3) it is explicit that approval was obtained from an ethics committee 0*/0/00* 0:00:00 AM.

**4.        Is there congruity between the stated philosophical perspective and research methodology?**

Well-designed qualitative studies should be based on particular philosophical perspective, thinking, principles, or set of shared beliefs that inform the meaning or interpretation of research data, as well as choice of methodology and methods. Some common philosophical perspectives are positivist, interpretivist, critical, and pragmatic (Kivunja & Kuyini, 2017). Following is a brief description and research methodologies suited for each one:

Positivist: Pursues an objective search for facts. Employs empirical or analytical approaches, including using deductive logic and formulation and testing of hypothesis to explore observations and answer questions. Suitable methodologies are usually quantitative; thus, studies based on this perspective would not be relevant for this review.

Interpretivist/constructivist: Understanding the subjective experience of the participants by interacting with them. Researcher values the need to understand individual rather than universal laws and the need to consider contextual factors. Suited methodologies, relevant to the review, include naturalist methodology, narrative inquiry, case study, grounded theory, phenomenology, hermeneutics, ethnography, phenomenography, action research, and inquiry.

Critical/transformative: Seeks to address power relationships set up within social structures and agency hidden by social practices. Critical perspective is about giving voice to the voiceless or less powerful and is interested in empowerment and removing oppressive structures around research participants. Researchers interact with participants while valuing respect for cultural norms. Suited methodologies, relevant to the review, include Neo-Marxist methodology, feminist theories, cultural studies, critical race theory, Freirean studies, participatory emancipation, postcolonial/indigenous methodology, queer theory, disability theories, action research.

Pragmatic: Emphasizes “workability” in research, i.e. the use of any research methods appropriate for addressing the research question without having to position oneself as positivist or interpretivist. Advocates the use of both qualitative and quantitative methods (i.e. mixed methods methodology). Thus, studies based on this perspective would not be relevant for this review.

To answer this question, consider the following: Does the report clearly state the methodological approach adopted on which the study is based? Does the report clearly state the philosophical or theoretical premises on which the study is based; or does the researcher provide a rationale for the chosen methodological approach, providing an insight into their thinking, beliefs, principles, values etc.? Is there congruence between the two (following the descriptions provided above)?

| **Answer YES, if** | **Answer NO, if** |
| --- | --- |
| - **Philosophical or theoretical premises of the study is are clearly presented, or text pertaining to the researcher’s thinking, beliefs, principles, values etc. is accessible by to the reader** - **The methodological approach to the study is clearly stated** - **There is congruence between the two** | - There is no statement on philosophical orientation, or pragmatic or positivist philosophical perspective is used. - There is no statement on the methodological approach to the study (i.e. only states that the study was qualitative or used qualitative methodology) - There is no congruence between the two |
| **Example: “IPA was chosen as it is committed to the detailed examination of the particular case, exploring how meaning is ascribed to participants’ experiences of interactions with the environment… with each [participant] purposefully and intensely analysed” (Opie, 2018, p.652). Methodology is explicit and the researcher’s values and thinking that forms the basis of the research (interpretivist/constructivist) was clearly presented, even though it wasn't explicitly stated. Both are also congruent.** | Example: Mayes (2014) states they used “a grounded theory approach … to understand data from the participants’ individual interviews”, in the data analysis section. But they do not explicitly state grounded theory as a methodology, nor is there any text pertaining to the researcher’s philosophical or theoretical perspective. The author does not provide a rationale as to why a grounded theory approach was adopted. |

**5.** **Is the influence of the researcher on the research, and vice-versa, addressed?**

This question is asking if the potential for the researcher to influence the study and for the potential of the research process itself to influence the researcher and her/his interpretations is acknowledged and addressed.

To answer this question, consider the following: Is the relationship between the researcher and the study participants addressed? Does the researcher critically examine her/his own role and potential influence during data collection? Is it reported how the researcher responded to events that arose during the study (i.e., any specific strategies utilized to limit or account for bias/influence)? This information can be found in the methods section of the paper. It may also be found in the limitations section of the discussion.

| **Answer YES, if** | **Answer NO, if** |
| --- | --- |
| **The researcher used one or more of the following strategies 0000-00-00 *0:00:*00 AM:**   - **Adequately and explicitly addressed the relationship between researcher and participants** - **Critically examined their own role, potential bias, and influence during the formulation of the research questions or data collection, including sample recruitment and choice of location; and/or during analysis and selection of data for presentation** - **Discussed how they responded to events during the study and whether they considered the implications of any changes in the research design** - **Employed field notes (reflective notes or memos) to record their personal reactions and biases after each interview/focus group** - **Made a conscious effort to follow rather than lead the direction of interviews/focus groups** - **Conducted member checking with participants** - **Maintained an audit trail** | - The potential for the researcher to influence the study, and vice-versa, is not addressed - Unable to locate any text specifically addressing relationship between the researcher and the study participants, researcher examining her/his own role and potential influence, or specific strategies utilized to limit or account for bias/influence - If text is present, the wording is unclear or confusing. |

**6.        Is there congruity between the research methodology and the methods used to collect data?**

This question considers whether the data collection methods are appropriate for the research methodology.

Qualitative studies use an open-ended format for their questions with a range of possible responses, when collecting data. Some examples of the data collection methods are in-depth or semi-structured interviews, focus groups, written or videotaped diaries, etc. Data collection is flexible and permits the addition, exclusion, or changes to the wording of particular interview questions. With these questions, participants are free to respond in their own words, and subsequent questions can be adjusted based on what is learned (Mack et al., 2005). This is in contrast to quantitative methods (surveys and some questionnaires) where the participants choose their responses from “closed-ended” or fixed categories (Mack et al., 2005).

To answer this question, consult the methods section of the study and consider if the authors clearly state their method of data collection. Did they describe the method? (*CASP Qualitative Checklist*, 2018). Is it evident that the data collection method is open-ended and flexible (e.g., focus group, semi-structured interviews, in-depth interviews etc.)? Are these approaches consistent with qualitative methodology (as stated previously)?

| **Answer YES, if** | **Answer NO, if** |
| --- | --- |
| - **Data were collected through a method consistent with qualitative research methodology (e.g., in-depth or semi-structured interviews, or focus groups)** - **There is flexibility in the data collection method (i.e., there is some spontaneity and adaptation of the interaction between the researcher and the study participant)** - **If text pertaining to the flexibility of data collection method is not presented (as in the example below), the use of in-depth, semi-structured interviews, or focus groups methods, as well as probe questions to elicit further exploration would also be appropriate** - **Questions are open-ended and participants are free to respond in their own words** | - The data collection method is not explicitly stated - Unable to locate any text specifically stating the method of data collection - If text is present, the wording is unclear or confusing. - Method of data collection has to be inferred by the reader by reading the rest of the paper. - Uses standardized, closed-ended surveys and questionnaires - The data collection method is very fixed from beginning to end - Uses structured interviews or open-ended questionnaires requiring written responses |
| **Example of flexibility in data collection: A general interview guide for the semi-structured interviews was created based on the researchers’ experiences in education and the review of the literature. Questions were added, subtracted, or modified based on the responses of the participants before and during the interview to allow for systematic, comprehensive interviewing (Mayes 2014, p.128).** |  |

**7.        Is there congruity between the research methodology and the representation and analysis of data?**

This question considers whether the data are analyzed and represented in ways that are congruent with the authors’ stated methodological position.

Qualitative data are generated in textual formats, usually in the forms of field notes, audio and video recordings, and transcripts (i.e., anything that is not quantitative/numerical) (Mack et al., 2005; Thorne, 2000). The analytical objective is to describe individual experiences and group norms as well as describe and explain relationships and variations for a particular phenomenon (Mack et al., 2005).

To answer this question, consider if the form of the data is clear (e.g., tape recordings, video materials, observational notes etc.)? Did the authors provide an in-depth description of their analysis process? Is it clear how categories/themes were derived from the data? (*CASP Qualitative Checklist*, 2018). Is the whole analysis process and its objective consistent with qualitative methodology (as described above)? Also consider if the authors follow a systematic, rigorous, and auditable process for data analysis (e.g., respondent validation, more than one analyst, peer debriefing, audit trail, triangulation of data or sources) (*CASP Qualitative Checklist*, 2018; McTavish et al., 2017; Thorne, 2000).

| **Answer YES, if** | **Answer NO, if** |
| --- | --- |
| - **The format of data generated is textual (obtained from audiotapes, videotapes, and field notes)** - **The authors provided in-depth descriptions of their analysis process, including how the data were reduced or transformed for analysis (McTavish et al., 2017)** - **It is clear how categories and themes were derived from raw data, where relevant** - **Uses constant comparative analysis, thematic analysis, narrative analysis, discourse analysis, hermeneutic analysis, ethnographic analysis, qualitative content analysis, or phenomenological approach (Thorne, 2000)** - **Overall objective of the analysis is to describe individual experiences, group norms, and/or describe and explain relationships and variations for a particular phenomenon** - **The authors followed a rigorous and auditable analytic process (Thorne, 2000)** - **They used one or more of the following strategies: member checking to see if participants agreed with the interpretations of the researcher, peers or consultants experienced in qualitative research reviewed the coding process, multiple analysts, peer debriefing, maintaining an audit trail, triangulation of data by using different data collection methods or sources, prolonged engagement (Irene Korstjens & Albine Moser, 2017; McTavish et al., 2017)** | - The format of data generated is numerical (obtained by assigning numerical values to responses) - In-depth descriptions of the analysis process is not provided or is unclear - It’s not clear how categories and themes were derived from raw data, where relevant - Objective of analysis is to quantify variation, predict causal relationships, or describe characteristics of a population (Mack et al., 2005) - A rigorous and auditable analytic process is not evident |
| **Example for qualitative data format: The semi-structured interviews were audio recorded and later transcribed (Opie, 2018, p. 653).**  **Example for in-depth description of the analysis process, including how categories and themes were derived: the stages used throughout the analysis were as follows: Transcripts were read and reread with notes made about important content and language. Further readings, coding notes and compiling categories resulted in the identification of emergent themes. Connections were then made between themes and a summary of pivotal themes created and placed in tentative categories (Opie, 2018, p.653).** | Example for unclear description of the analysis process, including how categories and themes were derived: claiming that the conceptual categories “emerged” from the data. |

**8.        Is there congruity between the research methodology and the interpretation of results?**

This question is asking whether the results are interpreted in ways that are appropriate for qualitative methodology.

Qualitative research findings do not speak for themselves. Instead, qualitative researchers give meaning to their findings by appropriately interpreting and structuring the data. As mentioned previously, qualitative researchers seek to explore or interpret meanings of experiences, actions, and/or beliefs of people (*CASP Qualitative Checklist*, 2018). Interpretation is basically transforming the data into meaningful and relevant findings (Thorne, 2000).

To answer this question, first consider if the findings are discussed in relation to the original research question or objective (*CASP Qualitative Checklist*, 2018). That is, were they interpreted in a meaningful way? To do this, read the results and discussion sections of the article. Then consider the robustness of their interpretation by examining the extent to which variation in data (i.e., participants’ experiences) was taken into account (*CASP Qualitative Checklist*, 2018).

| **Answer YES, if** | **Answer NO, if** |
| --- | --- |
| - **The researchers interpreted the data in a meaningful and relevant way** - **findings are discussed in relation to the original research question or objective, and are compared to past research** - **i.e., the authors put the new knowledge about phenomena and relations back into the context** - **The interpretation in results and discussion is robust** - **Variations in data were taken into account (e.g., mentioning an interpretation of an individual’s experience that seems to be different from the collective experience as a group)** | - The researchers did not interpret the data in a meaningful and relevant way - findings are not discussed in relation to the original research question or objective - The interpretation is not very robust - No mention of any variation in data or different individual experiences. |

**9.**  **Do the conclusions drawn from the research report flow from the analysis, or interpretation, of the data?**

This question concerns the relationship between the findings reported and the views or words of study participants. In appraising a paper, readers seek to satisfy themselves that the conclusions drawn by the researchers are grounded in the data (i.e., can be clearly linked to the text generated through observation, interviews or other processes) and that they do not only exist in the minds of the researchers.

To answer this question, thoroughly read the results, discussion, and conclusion. Then consider, does the conclusion overall seem to flow from the findings and the collected data?

| **Answer YES, if** | **Answer NO, if** |
| --- | --- |
| - **Overall, the conclusion seems to flow from the findings and collected data** - **The logical process used to develop findings is accessible (Thorne, 2000)** - **The relations between actual data and conclusions about data is explicit and appropriate (Thorne, 2000).** | - The conclusion doesn’t always seem to flow from the findings and collected data - It is not clear what logical process was followed to develop the findings - The relations between the actual data and conclusions about data is not explicit or appropriate |
