# Supplementary material for: Understanding school-based rehabilitation services through the lived experiences of children and youth with disabilities: a meta-aggregative review
Source: Front Public Health. 2026 Feb 27;14:1745224. doi: 10.3389/fpubh.2026.1745224 (PMC12982189; doi:10.3389/fpubh.2026.1745224)
Supplement: Supplementary file 4 [file Table_4.DOCX]

## **Appendix D**

*Extracted Findings, Supporting Illustrations, and Assigned Level of Credibility*

| **Category 1: Autonomy and agency at school and in therapy (13 findings)**  Children expressed a strong desire for control, choice, and influence in both their therapeutic experiences and broader participation at school. They valued being included in decisions about how and when therapy took place, the type and amount of information they received, and how their care was managed. Having opportunities to make choices, share preferences, and advocate for themselves helped foster a stronger sense of autonomy.  Autonomy refers to the ability to make independent decisions, while agency is the capacity to act on those decisions and influence outcomes. Both were important to the children. When therapy and school environments supported these concepts, children felt more informed, empowered, and in control. This increased sense of control helped reduce feelings of anxiety, fear, and frustration. | | | |
| --- | --- | --- | --- |
| Extracted Theme/Category | Verbatim or Paraphrased Description of Extracted Theme/Category | Supporting Illustrations | Level of Credibility |
| Treatment Values: Interference with daily living. (59) | Speech and language therapy had a varying influence on children’s daily lives. Some children considered speech and language therapy a waste of time. Children preferred to spend their time doing other things, for example, playing video games or watching movies. (p. 1532, clm 1, par 2, ln-1-5)      **If children received speech and language therapy during school time, they did not want to skip enjoyable classes like handcrafting classes or gymnastics.** (p. 1532, clm 2, par 1, ln 1-3) | **Participant1: I just think it is a waste of time. It is a waste of time.**    **Interviewer: Why do you experience this as a waste of time?**   **Participant 1: Because I have less time to play with my iPad.** (p. 1532, clm 1, par 2, ln 6-11)    **Participant 5: I did not like it if I was taken out of the class or the schoolyard for therapy.**  **Interviewer: Would you prefer that otherwise?**  **Participant 5: (Silence) After school or something like that.** (p. 1532, clm 2, par 1, ln 4-8) | Unequivocal |
| Treatment practices: Information flow(want information). (59) | **Some children valued receiving treatment-related information from SLPs, while others did not. Those who wanted information preferred it to be related to the structure of therapy sessions and the rationale behind therapy exercises.** (paraphrased from: p. 1532, clm 2, par 2, ln 1-5). | **Participant 5: I want to know what we are going to do. And why I need to do it [the exercises]. (**p. 1532, par 5, ln 6-7) | Unequivocal |
| Theme: Treatment practices: Information flow (don’t want information). (59) | **Some children did not find receiving treatment-related information from SLPs important (paraphrased from: p.** 1532, clm 2, par 2, ln 1-4) | **Participant 1: I don’t like it when she [the SLP] explains everything. It takes too much time.** (p. 1532, par 5, ln 8-9) | Unequivocal |
| Participating in the program had a physical and psychological impact: Psychological impact    Social experience. (Negative) (62) | The social experience was not universally positive; **two students reported preferring the company of their regular friends who they saw less of during the program. For one of these students, this resulted in a low attendance rate.** (p. 434, clm 2, par 3, ln 1-4) | **You have to exercise, but I want to play with my best mate. (Student)** (p. 434, clm 2, par 3, ln 5-6) | Unequivocal |
| The ideal supports are tailored, enabling and enriching, and enhance the child’s learning and social capital: Enabling. (65) | A further characteristic related to support which is enabling that is, delivered in a way which makes a child feel that they can succeed **Support that enables the child to make informed choices about their learning** One child for example, described having support to make good choices about books; books that would help them to become better at reading (paraphrased from: p.6, clm 2, par 1-3; p.7, clm 1, par 1). | **C1: “I want to pick books myself to read...help to choose a good book for me, not just what’s the reader, at school, we just do the readers”** (p.8, Table 5, Quote 10). | Credible |
| The ideal setting is one which is sentient, safe, inclusive and emancipatory: Power and control. (65) | Participants ideal setting was related to power and control. **Their ideal setting was one where the children had influence in decisions, and where they has more control over speaking in the class.** **In their ideal classroom, they would be given the space to use language for a variety of purposes rather than being restricted to answering the teacher’s questions.** This was in the context of recounting feelings of humiliation due to being unable to provide the right information requested by the teacher, in front of their peers In their ideal classroom, children would have more control over how language can be used and more opportunities to use language to think (paraphrased from; p. 9, clm 1, par3-4; P. 9, clm 2, par 1). | **C4: “Yeah, the kids are in charge in my school … they decide …”** (p. 9, Table 6, Quote 9)     **C1: “The teacher does all the talking and the children are not allowed talk- in this class the children can talk”**   (p. 9, Table 6, Quote 10)     C3: “… the teacher is always asking us for the answer … it annoys me the way the teacher asks a question that they know and you might not know it and you have to say ‘I don’t know’ and you act like a fool”  (p. 9, Table 6, Quote 11)     **C1: “In class … there is more time to talk... and more chances to practise talking cos... it helps you think about things”** (p. 9, Table 6, Quote 12) | Unequivocal |
| The child with DLD will be able to connect and participate, understand, self-manage and have a voice in their lives: Self-management. (65) | **A further outcome related to a child with DLD being able to independently manage their needs in school. All stakeholders discussed the importance of self-management, although they had differing views about the purpose of having such skills.** (p. 10, clm 1, par 4, ln 1-5.)     The children wanted strategies to manage complex/ nuanced issues related to navigating ethical dilemmas and peer relations. They also talked about the need for strategies that would help them to ‘survive’ and ‘stay safe’ in the context of their relationships with peers. For example, one child described developing an outer personality (which was contradictory to how they felt inside), to avoid being a target of bullying. (p.10 clm 2, par 3). | **C2: “… everybody thinks he is really brave but inside he is a really scared guy, he just acts like a tough guy in front of people … and they believe him and they leave him alone”**  (p. 10, Table 7, Quote 12) | Credible |
| Therapy Approaches Rooted in Conforming: Pathologizing Clinical Environments. (66) | Participants felt that the clinical environment of their therapy reinforced the idea that stuttering was a problem to be fixed rather than a natural part of their identity. While most described their speech therapists as kind and warm, the medicalized setting and focus of therapy made them feel as though their stuttering needed to be corrected. Some struggled to connect with their therapists due to the clinical setting. **Several participants wished they had more say in their treatment, particularly regarding how they were pulled out of class, which many found embarrassing and reinforcing of shame. One participant suggested that speech therapists foster autonomy by allowing children to choose how they arrive at therapy sessions.** (paraphrased from: p.105, clm 1, par 2-3) | **“I got put into speech therapy...**that means they thought there was a problem. (Auggie)  (p. 105, clm 1, par 2, ln 14-15)     “I wish I had had, you know, an SLP who just looked more like me. To be honest, it sounded more like me. I didn’t have any that stuttered, you know. I didn’t have any that even spoke with my particular dialect.” (Jason Jenya)  (p. 105, clm 1, par 2, ln 20-24)      **“It was [like] ‘You have to go with me right now’ and that I did not have a say” (Sophie)** (p. 105, clm 1, par 3, ln 4-5) | Unequivocal |
| Create Supportive Therapy Environments: Develop advocacy skills. (66) | **Participants felt that it was important to develop advocacy skills.** They expressed that it would have been helpful to problem-solve and practice difficult situations with their speech therapist. Participants believed it would have been beneficial to practice or role-play how to explain what stuttering is to others, tell teachers why presentations are hard, help others understand that stuttering varies, challenge stereotypes that stuttering is caused by nervousness, and practice self-disclosure. T**hey also mentioned that advocacy skills could help children understand and respond to listener reactions, allowing them to know how to react and what to say in negative social situations.** (paraphrased from: p.106, clm 1, par 1-2; p.106, clm 2, par 1) | **“navigate how awkward it is [and] figure out how to be okay with stuttering.” (Polly)** (p. 106, clm 1, par 3, ln 4-5)     **"if people didn’t like me for my stuttering, then that’s mainly their problem and not mine." (Casper)** (p. 106, clm 1, par 4, ln 6-7) | Credible |
| SLPs who work with CWS* should be patient and knowledgeable about stuttering. (67) | Participants wanted their speech therapists to understand the complexities of stuttering, but often they reported that their speech therapists did not have this knowledge. **Participants wanted therapists who were good listeners and who allowed them to make therapeutic choices based on what has and has not worked for them in the past.** (p. 7, par 1, ln 1-3) | "I think it’s just try and find out what that person needs by **um listening to them and um what’s um worked for them and um what hasn’t**, and um try to be um supportive um even if it’s hard for them" (CWS 16fc) (p. 7, par 1, ln 1-3)     "um I would say that you should let the kids who usually stutter a lot more to let them take their time you shouldn’t say it because that’s worse but you should let them talk, you should let them get their words out" (CWS14mc) (p. 6, Table 4, quote 1)  “but I never really had a speech therapist who really knew about stuttering and like in the past I’ve had therapists that would come with strategies to like change it or fix it and the thing is that stuttering does not have to be fixed at all.” (CWS16fa)  (p. 6, Table 4, quote 3) | Unequivocal |
| Discourse of behaviour. (69) | Children saw themselves as responsible for their own communication; they described speech-language pathology in terms of social expectations of others and saw co-operation as a matter of choice. **The children saw themselves as social actors with the power to make their own choices and determine their own behaviour.** (paraphrased from: p. 287, clm 1, par 3, ln 1-5; clm 2, par 4, ln 1-3) | I don’t like it. [...] I don’t want to. I don’t need any manners. [...] It’s just manners! [Declan] (p. 287, clm 1, par 3, ln 15-16) | Credible |
| Perceived Relevance of Acquired Knowledge: Perceived Relevance of Acquired Knowledge as a Complement to Exercise. (70) | **Participants highlight the complementarity between pain neuroscience education and the exercises and the relevance of pain neuroscience education on changing how they perceived exercise and its role in improving their NP.** (p.198, clm 2, par, 4, ln 1-4) | We have to try and remember everything we learned and say to ourselves: ok, no, this is chronic pain, **and I can control it, and do the exercises…**(Female, group 1) (p.198, clm 2, par, 4, ln 5-7) | Unequivocal |
| Perceived Relevance of Acquired Knowledge: Perceived Relevance of Acquired Knowledge as a Means to Facilitate Pain Reconceptualization and Management. (70) | The knowledge gained during the program was perceived as a facilitator of changes in feelings, attitudes, and behaviors toward pain. **Understanding the neurophysiological mechanisms underlying chronic pain altered how adolescents responded to pain, promoting more adaptive behaviors.** **These changes in attitude, feelings, and behaviors were associated with reduced anxiety, fear, and catastrophizing, as well as an increased sense of self-efficacy.** Participants collectively agreed on the significance of pain neuroscience education and its impact on their attitudes, feelings, and behaviors toward pain, along with **its influence on their ability to manage pain effectively.** | **Now we know that when the pain comes we do not have to stop [exercise/movement] … (Female, group 4)** (p.199, clm 1, par, 1, ln 7-8)     **The sessions helped us not to worry that much about pain, because that can bring anxiety and fear.** (Female, group 4) (p. 199, clm1, par 2, ln 4-5) | Unequivocal |
| **Category 2: Addressing and Validating Emotional Well-Being in Therapy (5 findings)**  Children valued therapy that supported their emotional well-being alongside their condition. They wanted therapists to create a safe space for open conversations about feelings, offer reassurance, and acknowledge the emotional impact of growing up with a condition. When their experiences were validated, children felt seen, accepted, and better able to cope and build confidence. In contrast, when emotional well-being was overlooked, children were often left feeling confused, frustrated, and alone in managing their emotions. | | | |
| Extracted Theme/Category | Verbatim or Paraphrased Description of Extracted Theme/Category | Supporting Illustrations | Level of Credibility |
| School characteristics: speech therapy. (64) | **Participants expressed a desire for more acknowledgment and discussions about stuttering, as well as involvement in support groups and emotional interventions.** They noted that therapy primarily focused on behavioral techniques, like reading aloud, and felt that addressing social and emotional aspects of stuttering could have been more beneficial later in life. **They emphasized the need for a more balanced therapeutic approach that addresses both socio-emotional needs and speech production, targeting speech, communication, and related thoughts and feelings.** (paraphrased from: p. 78, par 3, ln1-7; par 4, ln 1-2) | **Kenneth: One of the [school experience] themes would be certainly all the time wasted because I did not [pause], I did not address in any way during that time [in school] the emotional baggage that comes along with being a person who stutters. No therapist ever talked about it with me, at least not explicitly. And of course I could feel that emotional baggage but I never even thought about it. I just, I always was focused on the techniques, and it was all about the techniques, and the emotional side of it just wasn’t part of therapy. I wish I could take back some of that time. So that would be one of the themes, it would be absence of emotional therapy, and how it made the techniques close to useless, at least on a long-term basis.** (p.78, par 3, ln 8-14)    **Patricia: The system doesn’t address the unique individuality of each of their students. Ok. The system has one way of doing things, one mode of teaching style, one mode of discipline style, one mode, and it seems to cover most of the general population, except for a few kids here and there that might be emotionally different, that might be physically different, and if you stutter, it’s not an observable handicap.** (p.78, par 3, ln 15-18) | Unequivocal |
| Therapy Approaches Rooted in Conforming: Pressuring kids can backfire. (66) | Participants expressed feelings of helplessness at school due to a lack of participation options. **They were often required to engage in activities that made them uncomfortable, and adults did not recognize when they were not ready. Some described these situations as traumatic. One participant emphasized that therapists should consider both stress and safety when encouraging children to face their fears in communication.** (paraphrased from; p.105, clm 2, par 1, ln 1-11; P.105, clm 2, par 2) | **“Not everyone is ready to face the music quite yet. And you can traumatize someone trying to force them to swim by throwing them into the pool” (Jason Betterman).**  (p. 105, clm 2, par 1, ln 6-8)    We don’t actually build resilience through just repeated exposure. We actually just take on more damage.... **The helpful ways to grow are when we’re in a safe enough space and we’re moderately stressed and can deal with it, with a little bit of stress and a little bit of discomfort, but then have a safe space to return to and recharge in. (Huckleberry) (**p. 105, clm 2, par 1, ln 12-18) | Unequivocal |
| Create Supportive Therapy Environments: Help kids wade through confusion. (66) | **Participants highlighted the importance of making stuttering a discussable topic in speech therapy, emphasizing the need for children to explore their experiences in a supportive environment. Many participants shared that, as children, they did not have the opportunity to openly discuss their stuttering, leaving them to navigate their confusion on their own. Some participants recalled not initially recognizing their communication difficulties as stuttering, which led to feelings of frustration and uncertainty.** Furthermore, they expressed that, as children, they were unaware of coping strategies for stuttering other than simply trying to get rid of it. ne. Some participants shared that they did not initially recognize their communication challenges as stuttering, leading to frustration and uncertainty. They also expressed that they did not understand that there were options to cope with stuttering beyond trying to get rid of it.  (paraphrased from: p. 105, clm 2, par 4; p. 106, clm 1, par 1-2). | **“We would rather talk about it and then deal with the consequences that come with it than just put it under the rug.” (J.)** (p. 105, clm 2, par 4, ln 4-7)    I had no concept of what stuttering was. I had no idea why I stuttered. I just know that people told me to not do it or to slow down. And the more I tried, the harder it became." (Matthew) (p. 106, clm 1, par 1, ln 1-4)    **I think that’s where a lot of the shame comes in. It’s like what is wrong with me? ...I can talk perfectly fine on my own or to a dog. And as soon as it’s someone or in a situation where I’m worried about being judged then I can’t get the words out. (Bill)** (p. 106, clm 1, par 1, ln 10-14)    **“Kids will find their own solutions to problems if they just start talking about it.” (Jason Jenya) (**p. 106, clm 1, par 2, ln 10-11) | Unequivocal |
| Create Supportive Therapy Environments: Validate feelings: It's okay to stutter. (66) | **Participants emphasized the importance of speech therapists being responsive to the social and emotional aspects of stuttering. They highlighted that validating children's feelings and experiences is a crucial part of emotional support. Some participants expressed frustration with others minimizing their struggles and wished for genuine acknowledgment. Instead of having their difficulties dismissed, they wanted to hear that stuttering is okay and to be recognized for their efforts. They also emphasized the importance of reassurance, particularly in helping children understand that they can still achieve their goals while stuttering. Additionally, participants expressed the need for therapists to acknowledge that children are doing their best and to provide encouragement that their way of speaking is okay.** (paraphrased from: p. 106, clm 2, par 2-4) | **“if you don’t know how to help people with [the emotional] part...you’re going to hurt them.” (Rosalinda)** (p.106 clm 2, par 2 ln 3-5)    **“the way to help kids is to let them know that stuttering is okay...to encourage them to speak freely and easily.” (Daisy Duke) (**p.106 clm 2, par 2 ln 5-7)    **“Wow, this is really tough, and this is valid” (Hilton Silver).** (p.106 clm 2, par 3 ln 7-8)    “It’s so easy, I think, for people or kids to get in their head that idea [that] there’s something fundamentally wrong with them that needs to be fixed” (Owen). (p.106 clm 2, par 4 ln 3-5)    “[tell them] they can still be the captain of the tennis team and still stutter. You can still get a girlfriend and stutter, whatever you want.” (Jason Betterman) (p.106 clm 2, par 4 ln 11-14) | Unequivocal |
| CWS should be given agency in therapeutic goal setting: Feelings and attitudes about stuttering should be a focus. (67) | **Participants emphasized that addressing feelings and attitudes toward stuttering was important in their treatment.** (paraphrased from: p. 7, par 3, ln 1-2) | **“like teaching me like how to talk to a person about it or like you know like how to react when a person mentions it” (CWS08f)** (p. 7, par 3, ln 5)    "don’t help them get rid of their stuttering because but help them get comfortable for it because everyone’s gonna stutter once and a while" (CWS11f) (p. 6, Table 4, quote 8) | Unequivocal |
| **Category 3: A focus on fixing children in therapy is harmful. (6 findings)**  Children frequently internalized the belief that their disability or condition was a problem that needed to be fixed. This perception was reinforced through both direct and indirect messages in therapy such as being pulled out of class, the clinical setting, and the focus on correction and fluency techniques. Children expressed they wished they had been taught to accept their disability/condition as part of their identity instead of a problem that needed to be fixed or concealed. | | | |
| Extracted Theme/Category | Verbatim or Paraphrased Description of Extracted Theme/Category | Supporting Illustrations | Level of Credibility |
| Therapy Approaches Rooted in Conforming: Valuing fluency reinforces concealment. (66) | **Many participants stated that speech therapy approaches rooted in conformity were unhelpful and contributed to their concealment of stuttering. Fluency-shaping techniques, in particular, were described as harmful and damaging, with some participants noting that these methods increased the effort required for speaking and felt unnatural. Several recalled being explicitly taught strategies to hide their stuttering, such as singing words, using physical distractions, or resetting their speech. Praise for fluency reinforced the idea that stuttering should be avoided, leading some to feel pressure to please their therapists by concealing it. Additionally, participants expressed frustration with speech therapists who measured and counted moments of stuttering in front of them, describing these practices as discouraging and reinforcing the urge to hide their stuttering.** (paraphrased from; p.104-105, par 5-8.) | **“Gee, thank you for not stuttering. We like you so much better when you don’t stutter.” (Huckleberry)** (P. 104, clm 2, par 3, ln 3-7)    **“I’m not going to do that soft contact thing because it sounds stranger than stuttering.” (Bill)** (p. 104, clm 2, par 2, ln 11-12)    **“clicking away on little counters or something while the kid is speaking—that’s not going to be super helpful to being open about their stuttering.” (Hilton Silver)** (p. 104, clm 2, par 5, ln 3-6) | Unequivocal |
| Therapy Approaches Rooted in Conforming: Pathologizing Clinical Environments. (66) | **Participants felt that the clinical environment of their therapy reinforced the idea that stuttering was a problem to be fixed rather than a natural part of their identity. While most described their speech therapists as kind and warm, the medicalized setting and focus of therapy made them feel as though their stuttering needed to be corrected.** Some struggled to connect with their therapists due to the clinical setting. Several participants wished they had more say in their treatment, particularly regarding how they were pulled out of class, which many found embarrassing and reinforcing of shame. One participant suggested that speech therapists foster autonomy by allowing children to choose how they arrive at therapy sessions. (paraphrased from: p.105, clm 1, par 2-3) | “I got put into speech therapy**...that means they thought there was a problem**. (Auggie) (p. 105, clm 1, par 2, ln 14-15)    “I wish I had had, you know, an SLP who just looked more like me. To be honest, it sounded more like me. I didn’t have any that stuttered, you know. I didn’t have any that even spoke with my particular dialect.” (Jason Jenya) (p. 105, clm 1, par 2, ln 20-24)    “It was [like] ‘You have to go with me right now’ and that I did not have a say” (Sophie) (p. 105, clm 1, par 3, ln 4-5) | Unequivocal |
| SLPs who work with CWS* should be patient and knowledgeable about stuttering. (67) | Participants wanted their speech therapists to understand the complexities of stuttering, but often they reported that their speech therapists did not have this knowledge. Participants wanted therapists who were good listeners and who allowed them to make therapeutic choices based on what has and has not worked for them in the past. (p. 7, par 1, ln 1-3) | "I think it’s just try and find out what that person needs by um listening to them and um what’s um worked for them and um what hasn’t, and um try to be um supportive um even if it’s hard for them" (CWS 16fc) (p. 7, par 1, ln 1-3)    **"um I would say that you should let the kids who usually stutter a lot more to let them take their time you shouldn’t say it because that’s worse but you should let them talk, you should let them get their words out" (CWS14mc)** (p. 6, Table 4, quote 1)    **“**I have attended speech therapy probably ever since I could talk but I never really had a speech therapist who really knew about stuttering and like in the past **I’ve had therapists that would come with strategies to like change it or fix it and the thing is that stuttering does not have to be fixed at all** but um over time I met my good speech therapist Dana who’s very educated on stuttering” (CWS 16fa) (p. 7, par 2, ln 7-10) | Unequivocal |
| CWS should be given agency in therapeutic goal setting: Speech tools may eventually stop working. (67) | **Participants reported unfortunate treatment experiences with SLPs who lacked knowledge about stuttering and who focused exclusively on teaching fluency strategies.** (p. 7, par 2, ln 1-2) | **“So a lot of speech therapists are like obsessed with um using tools um and to me um once I like use a tool for like a year I almost get immune to it and then um my speech therapist keeps ranting on about like I need to use my tools more often then I open up and say like they don’t work anymore she just kind of says that I need to practice them more and then they will work so do not use tools as like your main punchline.”** (CWS12mb) (p. 7, par 2, ln 1-2) | Unequivocal |
| CWS should be given agency in therapeutic goal setting: Feelings and attitudes about stuttering should be a focus. (67) | Participants emphasized that addressing feelings and attitudes toward stuttering was important in their treatment. (paraphrased from: p. 7, par 3, ln 1-2) | “like teaching me like how to talk to a person about it or like you know like how to react when a person mentions it” (CWS08f) (p. 7, par 3, ln 5)    **"don’t help them get rid of their stuttering because but help them get comfortable for it because everyone’s gonna stutter once and a while" (CWS11f)** (p.6 Table 4, quote 8) | Unequivocal |
| Discourse of impairment. (69) | **Children described their SLCN in terms of deficits requiring treatment.** They compared their own communication to that of their peers, viewing their peers’ communication as the norm**. In doing so, many perceived their SLCN as a personal weakness. Rather than attributing their difficulties to social context, they located the problem within themselves**. In their accounts of speech-language pathology, children felt judged by others and followed prescribed activities under the initiative and control of others. This discourse of impairment positioned the therapist as an authority over them as clients. (paraphrased from: p. 285, clm 2, par 2, ln 1-6; p. 285, clm 2, par 3, ln 1-4; p. 286, clm 1, par 2, ln 1-4) | **Sometimes I find hard to remember easy words. [...] I’m not very good at words as well. Sometimes I try say something but it sometimes gets wrong and I can’t say it.** [William] (p. 285, clm 2, par 2, ln 12-15)    Do some work. Do some work. And do some work. And then when I finished I only do a few [shrugs] and then I go back to my class. [Ben] (p. 286, clm 1, par 2, ln 10-12) | Credible |
| ***Category 4: Children want Therapy That Is Relevant, Challenging, and Enjoyable (7 finding)***  Children described their ideal therapy experiences as those that felt relevant and interesting to them. Therapy was seen as more enjoyable and engaging when it was both appropriately challenging and fun. When therapists did not make therapy meaningful or stimulating, children reported feeling bored or disengaged during their sessions. For children participating in group therapy, the social component was something that encouraged engagement and that they enjoyed. | | | |
| Extracted Theme/Category | Verbatim or Paraphrased Description of Extracted Theme/Category | Supporting Illustrations | Level of Credibility |
| Treatment practices: Therapy content. (59) | **Children agreed that exercises should vary in difficulty. Most found word-level exercises “too easy” or “boring.” Older children viewed reading words and sentences with target consonants as less challenging.** (paraphrased from: p. 1532, clm 2, par 3, ln 1-7) | **Participant 5: I had to read a lot. While I was reading, I needed to focus on the speech sounds. I can already read, that was so boring.** (p. 1532, clm 2, par 3, ln 8-10) | Credible |
| Therapy practices: Confirmation and rewards. (59) | Some participants mentioned that their SLP used rewards, such as stickers, at the end of each therapy session, while others reported that they no longer received them**. Most children did not feel the need for rewards, and one participant noted that stickers did not make speech and language therapy more enjoyable.** (paraphrased from: p.1532 clm 2, par 5, ln 1-2 & p. 1533 clm 1, par 1, ln 1-6) | **Participant 1: I do not receive any rewards anymore.**  **Interviewer: Did you like speech and language therapy more when you did receive them?**  **Participant 1: No (laughs).** (p. 1533 clm 1, par 1, ln 7-10) | Unequivocal |
| Cultural hegemony and therapy-based activities as physical education. (Positive) (60) | **Results from activity 1 indicated that all participants enjoyed swimming and trampolining-related therapy. Their evident interest and enthusiasm suggest that these activities are enjoyable and suitable for the target group.** Through prolonged involvement with the school and pupils, it became clear that swimming and trampolining were used as ‘intensive intervention’ activities for students. (paraphrased from: p. 353, par 2, ln 1-6) | **Sophie: ‘I like … going swimming’.** (p.353, par 2, ln 3)    **Sophie: ‘I want to …go on the trampoline, I feel happy’** (p.353, par 3, ln 1-2)    **Theo: ‘I want to…jump on a trampoline’.** (p.353, par 3, ln 2-3) | Credible |
| Program factors contributed to positive outcomes: Attributes of program staff. (62) | The program staff were described as contributing to the success of the program. **The participants considered the following staff attributes as important: being collaborative, building rapport, being energetic and being open to the students’ ideas. The physiotherapy students who assisted with the program were described as “fun” and “excellent.”** (p. 344, clm 1, par 3, ln 1-6) | **Getting to run with (the physio student), I want to beat her! (Student)** (p. 344, clm 1, par 3, ln 7-8) | Credible |
| Participating in the program had a physical and psychological impact: Psychological impact. (Happiness, enjoyment and fun) (62) | The participants highlighted the psychological benefits of the program (p. 343, clm 1, par 6, ln 1-2).    **Students reported feeling happy, having fun, and being motivated as a result of the program** (p. 343, clm 1, par 6, ln 1-3**). They expressed enjoyment in the activities, describing the program as both challenging and motivating. The difficulty and variety of the tasks presented challenges, but students found motivation through encouragement and support in developing new skills and improving their fitness** (p. 343, clm 1, par 7, ln 1-4). | **Happy! It makes me feel good. (Student)** (p. 343, clm 1, par 6, ln 5)    **[My favourite thing was] working hard. I get really puffed out. (Student)** (p. 343, clm 1, par 7, ln 5-6) | Unequivocal |
| Theme: Participating in the program had a physical and psychological impact  Psychological impact: Social experience. (Positive) (62) | **The program was described as a positive social experience. Students said that they enjoyed spending time with other students and with the program staff.** (p. 343, clm 2, par 2, ln 1-3) | **[I liked] the friendships I made with the people at the program. (Student)** (p. 434, clm 2, par 2, ln 6-7) | Unequivocal |
| Perceived Adequacy of the Intervention. (70) | **The intervention was well accepted by all participants, was considered useful, and the materials and strategies used to implement it were deemed appropriate. Different words were used by participants to characterize it: “useful,” “relevant,” “interesting,” “beneficial,” “hard work,” “good,” “positive,” “excellent,” and “productive.” All participants considered the combination of pain neuroscience education and exercises as being appropriate, relevant, and complementary. (p.199, clm 2, par 5, ln 1-8) Participants pointed out the intelligibility of the language used to explain pain neuroscience** and the benefits of the group sessions opposed to one-to-one sessions, as this allowed them to know others in the same situation (shared experience) (p. 200, clm 1, par 1, ln 1-4) | **…it was important to combine theory, for us to learn more … as well as practice [exercises], so that we know small exercises that we can do at home … (Female, group 3** (p.199, clm 2, par 5, ln 9-11)    **I also think that the simplicity of the language used was important …, words were not very difficult, so that we keep it in our memory … (Female, group 3)** (p. 200, clm 1, par 1, ln 5-7)    [The sessions] in group I think it is better. We can share our ideas. Pains can be seen in very different ways. (Male, group 2) **(**p. 200, clm 1, par 1, ln 8-9) | Unequivocal |
| ***Category 5: Connecting with people who have similar lived experiences. (4 findings)***  Children emphasized wanting to connect with others who had similar experiences to their own. They expressed that having the opportunity to build relationships with peers and adults who understood what they were going through would help them feel less alone. Participants hoped that therapists would initiate and support these connections, allowing children a chance to make friends who shared their experiences. This desire for connection also extended to their therapists—one participant shared that he wished his therapist looked or sounded more like him, which would have helped him feel more understood and better connected during therapy. | | | |
| Extracted Theme/Category | Verbatim or Paraphrased Description of Extracted Theme/Category | Supporting Illustrations | Level of Credibility |
| Participating in the program had a physical and psychological impact: Psychological impact:  Social experience. (Positive) (62) | **The program was described as a positive social experience. Students said that they enjoyed spending time with other students** and with the program staff. (p. 343, clm 2, par 2, ln 1-3) | **[I liked] the friendships I made with the people at the program. (Student)** (p. 434, clm 2, par 2, ln 6-7) | Unequivocal |
| Therapy Approaches Rooted in Conforming: Pathologizing Clinical Environments. (66) | Participants felt that the clinical environment of their therapy reinforced the idea that stuttering was a problem to be fixed rather than a natural part of their identity. While most described their speech therapists as kind and warm, the medicalized setting and focus of therapy made them feel as though their stuttering needed to be corrected. **Some struggled to connect with their therapists due to the clinical setting.** Several participants wished they had more say in their treatment, particularly regarding how they were pulled out of class, which many found embarrassing and reinforcing of shame. One participant suggested that speech therapists foster autonomy by allowing children to choose how they arrive at therapy sessions. (paraphrased from: p.105, clm 1, par 2-3) | “I got put into speech therapy...that means they thought there was a problem. (Auggie) (p. 105, clm 1, par 2, ln 14-15)    **“I wish I had had, you know, an SLP who just looked more like me. To be honest, it sounded more like me. I didn’t have any that stuttered, you know. I didn’t have any that even spoke with my particular dialect.” (**Jason Jenya) (p. 105, clm 1, par 2, ln 20-24)    “It was [like] ‘You have to go with me right now’ and that I did not have a say” (Sophie) (p. 105, clm 1, par 3, ln 4-5) | Unequivocal |
| CWS should be given agency in therapeutic goal setting: Offer opportunities to connect with the community of PWS. (67) | **Participants also noted the importance of meeting and connecting with other stutterers.** (p. 7, par 3, ln 1) | **“ um probably meet a few people who stutter and like with other different speech impediments because it will probably help your patients feel better about their disability if you understand what they’re going through” (CWS17f)** (p. 7, par 3, ln 3-4) | Unequivocal |
| Perceived Adequacy of the Intervention. (70) | The intervention was well accepted by all participants, was considered useful, and the materials and strategies used to implement it were deemed appropriate. Different words were used by participants to characterize it: “useful,” “relevant,” “interesting,” “beneficial,” “hard work,” “good,” “positive,” “excellent,” and “productive.” All participants considered the combination of pain neuroscience education and exercises as being appropriate, relevant, and complementary. (p.199, clm 2, par 5, ln 1-8) **Participants pointed out** the intelligibility of the language used to explain pain neuroscience and **the benefits of the group sessions opposed to one-to-one sessions, as this allowed them to know others in the same situation (shared experience)** (p. 200, clm 1, par 1, ln 1-4) | …it was important to combine theory, for us to learn more … as well as practice [exercises], so that we know small exercises that we can do at home … (Female, group 3) (p.199, clm 2, par 5, ln 9-11)    I also think that the simplicity of the language used was important …, words were not very difficult, so that we keep it in our memory … (Female, group 3) (p. 200, clm 1, par 1, ln 5-7)    **[The sessions] in group I think it is better. We can share our ideas. Pains can be seen in very different ways. (Male, group 2) (p. 200, clm 1, par 1, ln 8-9)** | Unequivocal |
| ***Category 6: Navigating Peer Relationships and Social Inclusion at School (6 findings)***  Children expressed a desire for therapy and school staff to support their inclusion in everyday social situations, including how to initiate, respond to, and sustain interactions with peers. They emphasized the importance of learning how to self-disclose and advocate for themselves during challenging social encounters, including how to navigate self-disclosure and avoid bullying. | | | |
| Extracted Theme/Category | Verbatim or Paraphrased Description of Extracted Theme/Category | Supporting Illustrations | Level of Credibility |
| The ideal supports are tailored, enabling and enriching, and enhance the child’s learning and social capital: Relevant. (65) | **Children described their ideal support as relevant, meaning it is informed by their experiences of being socially excluded or unable to participate in school. Their ideal support would equip them with the skills to navigate the complexities of social contexts and relationships, allowing them to contribute in class. One child expressed the need for help in decoding the unwritten, unspoken rules in school—rules that others understood, but they did not. Other children mentioned the possibility of receiving assistance to improve their social status among peers, which would help them feel included**.  (paraphrased from; P. 8, clm 1, par 2-3) | **C3: “Yeah the rules just don’t make sense and also sometimes they (the teachers) say don’t have a phone in school and but they (children) do have a phone in school and they (the teachers) know it. I don’t get it … I want real help with understanding the rules that can be broken”** (p. 8, Table 5, Quote 16)    **C2: “I want to be cool... for others to think I’m cool … so they will want to play with me … can someone learn me that?”** (p. 8, Table 5, quote 17). | Unequivocal |
| The child with DLD will be able to connect and participate, understand, self-manage and have a voice in their lives: Connect and participate. (65) | **Children emphasized the importance of developing social skills to build and maintain friendships, highlighting the need to learn the language of their peers as a way to connect.** They also valued participation in class, not as a way to demonstrate knowledge but as an opportunity to contribute ideas and learn through language. Their perspective on participation differed from that of adults, as they saw it as a means of engagement and collaboration rather than simply answering questions. (p. 9, clm 2, par 2-4; p. 10, clm 1, par 1) | **C1: “I want to talk, you know like, talking the way they (peers) do, so they will listen and think I’m interesting**” (p. 10, Table 7, Quote 3)    C1: “to be able to talk more in class, so I can to try out new ideas” (p. 10, Table 7, Quote 5) | Unequivocal |
| The child with DLD will be able to connect and participate, understand, self-manage and have a voice in their lives: Understand. (65) | **An important outcome described by children was the ability to understand and make inferences about people and social situations. They wanted to be able to read others and grasp the unwritten rules of school, which they found difficult to comprehend. Their current struggle with this often led to feelings of exclusion from the school community.** (paraphrased from; P. 10, clm 1, par 2-3). | **C2: “… to be able to listen to people’s thoughts and see inside their head”** (p. 10, Table 7, Quote 7)    **C6: “if this person was feeling this way … knowing how that person is feeling … learning what would you do”** (p. 10, Table 7, Quote 8) | Unequivocal |
| The child with DLD will be able to connect and participate, understand, self-manage and have a voice in their lives: Self-management. (65) | A further outcome related to a child with DLD being able to independently manage their needs in school. All stakeholders discussed the importance of self-management, although they had differing views about the purpose of having such skills. (p. 10, clm 1, par 4, ln 1-5.)     **The children wanted strategies to manage complex/ nuanced issues related to navigating ethical dilemmas and peer relations. They also talked about the need for strategies that would help them to ‘survive’ and ‘stay safe’ in the context of their relationships with peers. For example, one child described developing an outer personality (which was contradictory to how they felt inside), to avoid being a target of bullying.** (p.10 clm 2, par 3). | **C2: “… everybody thinks he is really brave but inside he is a really scared guy, he just acts like a tough guy in front of people … and they believe him and they leave him alone”** (p. 10, Table 7, Quote 12) | Credible |
| Create Supportive Therapy Environments: Develop advocacy skills. (66) | Participants felt that it was important to develop advocacy skills. **They expressed that it would have been helpful to problem-solve and practice difficult situations with their speech therapist. Participants believed it would have been beneficial to practice or role-play how to explain what stuttering is to others, tell teachers why presentations are hard, help others understand that stuttering varies, challenge stereotypes that stuttering is caused by nervousness, and practice self-disclosure.** T**hey also mentioned that advocacy skills could help children understand and respond to listener reactions, allowing them to know how to react and what to say in negative social situations.** (paraphrased from: p.106, clm 1, par 1-2; p.106, clm 2, par 1) | **“navigate how awkward it is [and] figure out how to be okay with stuttering.” (Polly)** (p. 106, clm 1, par 3, ln 4-5)     **"if people didn’t like me for my stuttering, then that’s mainly their problem and not mine." (Casper)** (p. 106, clm 1, par 4, ln 6-7) | Credible |
| CWS should be given agency in therapeutic goal setting: Feelings and attitudes about stuttering should be a focus. (67) | **Participants emphasized that addressing feelings and attitudes toward stuttering was important in their treatment.** (paraphrased from: p. 7, par 3, ln 1-2) | **“like teaching me like how to talk to a person about it or like you know like how to react when a person mentions it” (CWS08f)** (p. 7, par 3, ln 5)    "don’t help them get rid of their stuttering because but help them get comfortable for it because everyone’s gonna stutter once and a while" (CWS11f) (p. 6, Table 4, quote 8) | Unequivocal |
| ***Category 7: Therapy is a Space to Learn and Practice New Skills (3 findings)***  Children viewed therapy as a place to learn, practice, and improve important skills. They expected therapy to help them develop abilities like clearer communication or physical skills through practice and support. Therapy was valued when it offered consistent opportunities for skill-building, encouraged effort, and treated mistakes as part of the normal learning process. | | | |
| Extracted Theme/Category | Verbatim or Paraphrased Description of Extracted Theme/Category | Supporting Illustrations | Level of Credibility |
| Treatment Values: Expectations and emotions around therapy. (Positive) (59) | Some children (participants 2, 3 and 5) had clear expectations around speech and language therapy: **they told that they needed to go to the SLP to ‘help’ their speech. They explained that they also expected that practising with the SLP would make their speech more understandable to others.** (p. 1531, clm 2 par 2, ln 1-6) | **Participant 5: Some children don’t understand me. I hope practising will help me with that.** (p. 1532, clm 1, ln 1-4) | Unequivocal |
| Factors related to social support: Professional support. (61) | Our participants not only identified physiotherapists as experts in PA but also recognized them as a source of support as they were growing up. Most participants attending special education schools described their experiences with physiotherapy as positive and were upset that such treatments were no longer available. (p.6656, clm 2, par 3, ln 1-5) | The physiotherapists were there for me. They were determined and motivated for my own good. **I got new exercises every time, that’s why I really liked going to physiotherapy.**  Harvey (20, III, SE) (p.6654, clm 1, par 3, ln 5-7) | Credible |
| Discourse of learning. (69) | **Children described communication as a skill to be practiced and learned, with speech-language pathology as one means of achieving this.** They framed their weaknesses as the ordinary challenges of remembering, making an effort, and learning. **Over time, they saw opportunities for improvement as they practiced and mastered new skills, with both their own efforts and the support of those who empowered them by teaching them how to do things. The learning discourse allowed children to describe themselves as solving communication problems through their own efforts, improving their skills with practice, recognizing their mistakes and correcting them, and learning how to do new things.** (p. 287, clm 2, par 4, ln 1-3; (paraphrased from: p. 288, clm 1, par 2, ln 1-5; (p. 288, clm 2, par 2, ln 1-5) | **I don’t really care they mistake me on that. Cos that don’t really matter, do it? You just need to try and learn it. [. . .] You just need to learn what you done, and you just need to carry on. [Harry]** (p. 287, clm 1, par 1, ln 10-13) | Unequivocal |
| ***Category 8: Lack of Access to Services After School (2 findings)***  Children mentioned that when they got older or aged out of school that services became inaccessible to them and had negative consequences on their lives. | | | |
| Extracted Theme/Category | Verbatim or Paraphrased Description of Extracted Theme/Category | Supporting Illustrations | Level of Credibility |
| Cultural hegemony and therapy-based activities as physical education. (Negative) (60) | An older pupil expressed a desire to re-engage in trampolining, a therapy activity that was no longer accessible to Sixth Form students. His positive memories of past participation reflected his interest in continuing the activity. **Older participants wished to continue therapy activities they had enjoyed before entering Sixth Form, but these sessions were no longer available through their school.** Decisions made by those in power have restricted pupils' access to the physical activities they previously enjoyed once they enter Sixth Form education. (p. 535, par 5, ln 1-10) | **Jack: ‘I want to … go on the trampoline’.** (p. 535, par 5, ln 4) | Credible |
| Availability of relevant PA and professional guidance. (61) | **Physiotherapy also was not available for young people**  (p.6653, clm 2, par 7, ln 1) | **“I don’t move a lot. And there is a reason for that. After I left school the physiotherapy treatments stopped. I had to wait months to get 12 sessions of treatment from the health insurance, my muscles weakened….”** Harvey (20, III, SE)  (p.6653, clm 2, par 7, ln 2-5) | Credible |
| ***Category 9: Therapy Should Align with the School Schedule (4 findings)***  Children expressed frustration with being pulled out of class for therapy, particularly when it meant missing activities they enjoyed or considered important. They emphasized that therapy should not interfere with learning, socializing, or personal interests. Additionally, children noted that the timing of sessions mattered, therapy scheduled late in the day or week often left them feeling tired, reducing their ability to participate fully in classroom activities afterward. | | | |
| Extracted Theme/Category | Verbatim or Paraphrased Description of Extracted Theme/Category | Supporting Illustrations | Level of Credibility |
| Treatment Values:  Interference with daily living. (59) | Speech and language therapy had a varying influence on children’s daily lives. Some children considered speech and language therapy a waste of time. Children preferred to spend their time doing other things, for example, playing video games or watching movies. (p. 1532, clm 1, par 2, ln-1-5)      **If children received speech and language therapy during school time, they did not want to skip enjoyable classes like handcrafting classes or gymnastics.** (p. 1532, clm 2, par 1, ln 1-3) | **Participant1: I just think it is a waste of time. It is a waste of time.**    **Interviewer: Why do you experience this as a waste of time?**   **Participant 1: Because I have less time to play with my iPad.** (p. 1532, clm 1, par 2, ln 6-11)    **Participant 5: I did not like it if I was taken out of the class or the schoolyard for therapy.**  **Interviewer: Would you prefer that otherwise?**  **Participant 5: (Silence) After school or something like that.** (p. 1532, clm 2, par 1, ln 4-8) | Unequivocal |
| Program factors contributed to positive outcomes: School setting. (62) | Participants felt that the program naturally fit into the school setting and was seen as an extension of school activities. **They appreciated that it ran during lunchtime, a time when students would have otherwise been sedentary, and it allowed them to avoid missing classes.** The school setting was considered ideal due to its dedicated spaces for exercise and available equipment. However, participants noted that crowding, particularly in indoor areas during rainy weather, was a challenge for the program. (paraphrased from: p. 343, clm 2, par 5-7; p. 344, clm 1, par 1-2) | **I think it was good to have it at lunchtime,** but also bad because on wet days everyone’s in crowded in one spot... I don’t like that [it’s] messy, everything happening. I like to focus on task at hand. (Student) (p.344, clm 1, par 2, ln 3-6) | Unequivocal |
| Participating in the program had a physical and psychological impact: Physical impact of exercise. (Negative) (62) | **There was discussion that students could experience fatigue immediately after the exercise sessions. This could have a negative impact on afternoon classes or at home that evening. The program was seen as an additional activity the students needed to find energy for, in the context of an already busy school schedule. Students talked about pushing themselves during the exercise sessions, despite knowing that they might feel tired later on.** (p. 343, clm 1, par 3, ln 1-8) | **Finding energy sometimes, especially on Fridays, [was hard]. (Student)** (p. 343, clm 1, par 3, ln 9-10) | Credible |
| Therapy Approaches Rooted in Conforming: Pathologizing Clinical Environments. (66) | Participants felt that the clinical environment of their therapy reinforced the idea that stuttering was a problem to be fixed rather than a natural part of their identity. While most described their speech therapists as kind and warm, the medicalized setting and focus of therapy made them feel as though their stuttering needed to be corrected. Some struggled to connect with their therapists due to the clinical setting. **Several participants wished they had more say in their treatment, particularly regarding how they were pulled out of class, which many found embarrassing and reinforcing of shame. One participant suggested that speech therapists foster autonomy by allowing children to choose how they arrive at therapy sessions.** (paraphrased from: p.105, clm 1, par 2-3) | “I got put into speech therapy...that means they thought there was a problem. (Auggie) (p. 105, clm 1, par 2, ln 14-15)     “I wish I had had, you know, an SLP who just looked more like me. To be honest, it sounded more like me. I didn’t have any that stuttered, you know. I didn’t have any that even spoke with my particular dialect.” (Jason Jenya) (p. 105, clm 1, par 2, ln 20-24)      **“It was [like] ‘You have to go with me right now’ and that I did not have a say” (Sophie)** (p. 105, clm 1, par 3, ln 4-5) | Unequivocal |
| ***Category 10: Therapy should support and promote participation at school (6 findings)***  Children valued therapy that enabled them to participate more fully in school life. While the meaning of participation varied for each child, it often included being included in classroom activities, engaging with peers, and feeling like an active member of the school community. When there was a lack of participation options at school children felt scared or unable to participate in school activities and interact with peers. Therapists and school staff should support and encourage participation at school. | | | |
| Extracted Theme/Category | Verbatim or Paraphrased Description of Extracted Theme/Category | Supporting Illustrations | Level of Credibility |
| Participating in the program had a physical and psychological impact: Physical impact of exercise (Positive) (62) | Participants believed that the program resulted in physical benefits. **Both students and their parents noted improved mobility, with better walking quality and increased walking distance after the program. Participants also felt that students' aerobic capacity improved, enabling them to engage in activities, such as playing games, for longer periods**. (paraphrased from: p. 342, clm 2, par 3, ln 1-5; p. 343, clm 1, par 1, ln 1-3) | **I can easily now run faster. (Student)** (p. 343, clm 1, ln 1)  **I feel more energetic. And I have more stamina to do things with my friends, like running around in the playground. (Student)** (p. 343, clm 1, par 1, ln 6-7) | Unequivocal |
| Theme Related to School Participation and Accommodations (63) | **Participation in class continued to be a concern for some students who stutter, and some will try and avoid speaking situations.** Some students benefited from accommodations, which were specific to each student.  (p. 1332, clm 2, par 1, ln 1-4) | Participant 7: “**I try to avoid public speaking**. **When I present, most of my teachers let me go after school when the students are not there. I don’t do it all of the time because sometimes I have the courage to go up and do it. If I feel like I can do it, then I want to do it. When I am reading in class, my teacher lets me go up and read in private. My speech therapist at school when I had a presentation in my English class, she talked with my English teacher and said that if I could pick a couple of friends to come and listen to the presentation. Maybe not all of them but I do remember most of them being helpful towards anything.”** (p. 1332, clm 2, Quote 7) | Unequivocal |
| Participants’ personal reflections/observations of school: suggestions for schoolteachers and speech-language pathologists. (64) | Though some participants perceived improvements, other participants felt that aspects of the school climate were still problematic for PWS. (p. 79, par 4, ln 4-5).    This suggests that PWS may still be more vulnerable to teasing experiences. (p. 79, par 4, ln 7-8) | Dena: But I think for young children. . . they need some relaxation methods, they need to use some cognitive restructuring methods, they need to use some desensitization methods, they probably need to be, to practice talking, **I think if you catch them at a very young age, and you get them to participate in activities where they have to talk more, and to encourage them to talk, I think that would be very important.** (p. 79, par 3, ln 2-6) | Credible |
| The ideal supports are tailored, enabling and enriching, and enhance the child’s learning and social capital: Relevant. (65) | **Children described their ideal support as relevant, meaning it is informed by their experiences of being socially excluded or unable to participate in school. Their ideal support would equip them with the skills to navigate the complexities of social contexts and relationships, allowing them to contribute in class.** One child expressed the need for help in decoding the unwritten, unspoken rules in school—rules that others understood, but they did not. Other children mentioned the possibility of receiving assistance to improve their social status among peers, which would help them feel included.  (paraphrased from; P. 8, clm 1, par 2-3) | C3: “Yeah the rules just don’t make sense and also sometimes they (the teachers) say don’t have a phone in school and but they (children) do have a phone in school and they (the teachers) know it. I don’t get it … I want real help with understanding the rules that can be broken” (p. 8, Table 5, Quote 16)    **C2: “I want to be cool... for others to think I’m cool … so they will want to play with me … can someone learn me that?”** (p. 8, Table 5, quote 17). | Unequivocal |
| The child with DLD will be able to connect and participate, understand, self-manage and have a voice in their lives: Connect and participate. (65) | Children emphasized the importance of developing social skills to build and maintain friendships, highlighting the need to learn the language of their peers as a way to connect. **They also valued participation in class, not as a way to demonstrate knowledge but as an opportunity to contribute ideas and learn through language. Their perspective on participation differed from that of adults, as they saw it as a means of engagement and collaboration rather than simply answering questions**. (p. 9, clm 2, par 2-4; p. 10, clm 1, par 1) | C1: “I want to talk, you know like, talking the way they (peers) do, so they will listen and think I’m interesting” (p. 10, Table 7, Quote 3)    **C1: “to be able to talk more in class, so I can to try out new ideas”** (p. 10, Table 7, Quote 5) | Unequivocal |
| Therapy Approaches Rooted in Conforming: Pressuring kids can backfire. (66) | **Participants expressed feelings of helplessness at school due to a lack of participation options.** They were often required to engage in activities that made them uncomfortable, and adults did not recognize when they were not ready. Some described these situations as traumatic. One participant emphasized that therapists should consider both stress and safety when encouraging children to face their fears in communication. (paraphrased from; p.105, clm 2, par 1, ln 1-11; p.105, clm 2, par 2) | **“Not everyone is ready to face the music quite yet. And you can traumatize someone trying to force them to swim by throwing them into the pool” (Jason Betterman).** (p. 105, clm 2, par 1, ln 6-8)    We don’t actually build resilience through just repeated exposure. We actually just take on more damage.... The helpful ways to grow are when we’re in a safe enough space and we’re moderately stressed and can deal with it, with a little bit of stress and a little bit of discomfort, but then have a safe space to return to and recharge in. (Huckleberry) (p. 105, clm 2, par 1, ln 12-18) | Unequivocal |
| ***Category 11: Tailoring Needs and Accommodations to Individual Children (7 findings)***  School support should be personalized to each child’s unique and evolving needs. This includes therapists and teachers adapting their approaches based on what will best support a child’s participation, comfort, and well-being on any given day. It also meant having a flexible approach in therapy to address the individual needs of children. Children emphasized the importance of flexibility, highlighting that their needs can change over time or even from moment to moment.  When accommodations and participation options were not available, then children felt feelings of anxiety and fear at school. | | | |
| Extracted Theme/Category | Verbatim or Paraphrased Description of Extracted Theme/Category | Supporting Illustrations | Level of Credibility |
| Treatment practices: Therapy dosage. (59) | **Some children considered 30 min of speech and language therapy as too long, while others did not. Children who told that 30 min was long also explained that this feeling faded once the therapy started**. (p. 1533, clm 1 par 3, ln 1-4) | **Participant 1: Speech and language therapy lasts long. But when it [the session] is over, I think ‘Wow, that went by quickly.** (p. 1533, clm 1 par 3, ln 4-6) | Credible |
| Theme Related to School Participation and Accommodations. (63) | **Participation in class continued to be a concern for some students who stutter, and some will try and avoid speaking situations.** Some students benefited from accommodations, which were specific to each student. (p. 1332, clm 2, par 1, ln 1-4) | Participant 7: “**I try to avoid public speaking**. **When I present, most of my teachers let me go after school when the students are not there. I don’t do it all of the time because sometimes I have the courage to go up and do it. If I feel like I can do it, then I want to do it. When I am reading in class, my teacher lets me go up and read in private. My speech therapist at school when I had a presentation in my English class, she talked with my English teacher and said that if I could pick a couple of friends to come and listen to the presentation. Maybe not all of them but I do remember most of them being helpful towards anything.”** (p. 1332, clm 2, Quote 7) | Unequivocal |
| School characteristics: Speech therapy. (64) | Participants expressed a desire for more acknowledgment and discussions about stuttering, as well as involvement in support groups and emotional interventions**.** They noted that therapy primarily focused on behavioral techniques, like reading aloud, and felt that addressing social and emotional aspects of stuttering could have been more beneficial later in life. **They emphasized the need for a more balanced therapeutic approach that addresses both socio-emotional needs and speech production, targeting speech, communication, and related thoughts and feelings.** (paraphrased from: p. 78, par 3, ln1-7; par 4, ln 1-2) | Kenneth: One of the [school experience] themes would be certainly all the time wasted because I did not [pause], I did not address in any way during that time [in school] the emotional baggage that comes along with being a person who stutters. No therapist ever talked about it with me, at least not explicitly. And of course I could feel that emotional baggage but I never even thought about it. I just, I always was focused on the techniques, and it was all about the techniques, and the emotional side of it just wasn’t part of therapy. I wish I could take back some of that time. So that would be one of the themes, it would be absence of emotional therapy, and how it made the techniques close to useless, at least on a long-term basis. (p.78, par 3, ln 8-14)    **Patricia: The system doesn’t address the unique individuality of each of their students. Ok. The system has one way of doing things, one mode of teaching style, one mode of discipline style, one mode, and it seems to cover most of the general population, except for a few kids here and there that might be emotionally different, that might be physically different, and if you stutter, it’s not an observable handicap.** (p.78, par 3, ln 15-18) | Unequivocal |
| The ideal supports are tailored, enabling and enriching, and enhance the child’s learning and social capital: Enriching. (65) | **Participants described the importance of providing enriching learning opportunities that are not delivered through language instruction alone. These may occur in or out of the classroom.** (paraphrased from; P. 7, clm 1, par 2; P.7 clm 2, par 1, ln 1-2) | **C1: “I like experiments it makes it easy to learn if you are doing it. So we did an experiment before with washing up liquid and more art cos you can think about things, it’s another, it gives you another way to think about things”** (p.8, Table 5, Quote 11). | Unequivocal |
| The ideal supports are tailored, enabling and enriching, and enhance the child’s learning and social capital: Individually tailored. (65) |  | **C5: “I can do the work it’s hard but not too hard … so I’m learning … but I feel good”** (p. 8, Table 5, Quote 3)    **C4: “... I like learning about the past. I want help with hard stuff that I like, like that”** (p. 8, Table 5, Quote 5) | Credible |
| Therapy Approaches Rooted in Conforming: Pressuring kids can backfire. (66) | **Participants expressed feelings of helplessness at school due to a lack of participation options. They were often required to engage in activities that made them uncomfortable, and adults did not recognize when they were not ready. Some described these situations as traumatic. One participant emphasized that therapists should consider both stress and safety when encouraging children to face their fears in communication.** (paraphrased from; p.105, clm 2, par 1, ln 1-11; P.105, clm 2, par 2) | **“Not everyone is ready to face the music quite yet. And you can traumatize someone trying to force them to swim by throwing them into the pool” (Jason Betterman).** (p. 105, clm 2, par 1, ln 6-8)    We don’t actually build resilience through just repeated exposure. We actually just take on more damage.... **The helpful ways to grow are when we’re in a safe enough space and we’re moderately stressed and can deal with it, with a little bit of stress and a little bit of discomfort, but then have a safe space to return to and recharge in. (Huckleberry)** (p. 105, clm 2, par 1, ln 12-18) | Unequivocal |
| Create Supportive Therapy Environments: Help kids wade through confusion. (66) | Participants highlighted the importance of making stuttering a discussable topic in speech therapy, emphasizing the need for children to explore their experiences in a supportive environment. Many participants shared that, as children, they did not have the opportunity to openly discuss their stuttering, leaving them to navigate their confusion on their own. Some participants recalled not initially recognizing their communication difficulties as stuttering, which led to feelings of frustration and uncertainty. Furthermore, they expressed that, as children, they were unaware of coping strategies for stuttering other than simply trying to get rid of it.**Some participants shared that they did not initially recognize their communication challenges as stuttering, leading to frustration and uncertainty. They also expressed that they did not understand that there were options to cope with stuttering beyond trying to get rid of it.** (paraphrased from: p. 105, clm 2, par 4; p. 106, clm 1, par 1-2). | “We would rather talk about it and then deal with the consequences that come with it than just put it under the rug.” (J.)  (p. 105, clm 2, par 4, ln 4-7)    **I had no concept of what stuttering was. I had no idea why I stuttered. I just know that people told me to not do it or to slow down. And the more I tried, the harder it became." (Matthew) (**p. 106, clm 1, par 1, ln 1-4)    I think that’s where a lot of the shame comes in. It’s like what is wrong with me? ...I can talk perfectly fine on my own or to a dog. And as soon as it’s someone or in a situation where I’m worried about being judged then I can’t get the words out. (Bill)  (p. 106, clm 1, par 1, ln 10-14)    **“Kids will find their own solutions to problems if they just start talking about it.” (Jason Jenya) (p. 106, clm 1, par 2, ln 10-11)** | Unequivocal |
| ***Category 12: Therapists should be advocates for children (2 findings)***  Children expressed a desire for therapists to take on advocacy roles within the school setting. They appreciated when therapists helped ensure their needs were understood and supported by teachers, peers, and other school staff. Advocacy included educating others about the child’s condition and needs, promoting awareness, and fostering greater understanding within the school community. | | | |
| Extracted Theme/Category | Verbatim or Paraphrased Description of Extracted Theme/Category | Supporting Illustrations | Level of Credibility |
| Create safe school environments: Educating others. (66) | **Participants emphasized the need for adults in schools to understand stuttering and provide support. They believed all staff should be educated on the variability of stuttering, the loss of control it involves, and why advice like slow down or take a breath is unhelpful. Educating school staff, parents, and peers was seen as crucial, with suggestions that speech therapists provide training in meetings or one-on-one discussions.** Classroom presentations were also recommended to help peers understand stuttering, but participants stressed that these should be child-driven, allowing the child to decide if and how they want to share their experiences. While presentations could be beneficial, they could also be harmful if not handled with care and without ensuring the child’s autonomy in the process. (paraphrased from: p. 107, clm 2, par 2-3) | **“[showing] there are other people who know and who get it, who [they] can find support in. I think that would be really big” (Felix Felipe).** (p. 107, clm 2, par 2, ln 10-13) | Credible |
| The leadership of the support group: Physiotherapists’ role regarding peer support. (71) | Previous learners with paraplegia expected the school’s physiotherapist to provide guidance to the peer-group leader by being an approachable source of knowledge, teaching peer supporters, assisting with occasionally giving input and receiving feedback from the group sessions. **In addition, they hoped physiotherapists would advocate for their needs at the school management level (**p. 7, clm 1, par 2, ln 1-7) | … there should be the likes of … the help of the hospital physiotherapist, and the school, and other special schools, we will manage.’ (PL2, female, 23 years) (p.7, clm 1 par 2, ln 8-10)    **If one person could go or two persons, and they represent the paraplegics, and they would talk with someone from physio.’ (PL3, female, 26 years)** (p.7, clm 1, par 2, ln 11-13)    They should raise this by someone … maybe, the physio-people.’ (PL3, female, 26 years) (p. 7,  clm 1, par 2, ln 14-15) | Unequivocal |
| **Category 13: The Importance of Knowledgeable, Supportive and Empathetic Therapists (7 findings)**  Children valued therapists who were knowledgeable, supportive, empathetic, and approachable. A warm and understanding environment made children feel comfortable sharing their challenges and motivated them to participate in therapy. They also appreciated therapists who understood disability and could offer informed treatment options. In contrast, a lack of empathy or knowledge left children feeling frustrated, unheard, and invalidated. | | | |
| Extracted Theme/Category | Verbatim or Paraphrased Description of Extracted Theme/Category | Supporting Illustrations | Level of Credibility |
| Factors related to social support: Professional support. (61) | Our participants not only identified physiotherapists as experts in PA but also **recognized them as a source of support as they were growing up**. Most participants attending special education schools described their experiences with physiotherapy as positive and were upset that such treatments were no longer available. (p.6656, clm 2, par 3, ln 1-5) | **The physiotherapists were there for me. They were determined and motivated for my own good.** I got new exercises every time, that’s why I really liked going to physiotherapy. Harvey (20, III, SE) (p.6654, clm 1, par 3, ln 5-7) | Credible |
| Participating in the program had a physical and psychological impact: Psychological impact: Happiness, enjoyment and fun. (Positive) (62) | The participants highlighted the psychological benefits of the program (p. 343, clm 1, par 6, ln 1-2).    **Students reported feeling happy, having fun, and being motivated as a result of the program** (p. 343, clm 1, par 6, ln 1-3).    They expressed enjoyment in the activities, describing the program as both challenging and motivating. **The difficulty and variety of the tasks presented challenges, but students found motivation through encouragement and support in developing new skills and improving their fitness** (p. 343, clm 1, par 7, ln 1-4). | **Happy! It makes me feel good. (Student)** (p. 343, clm 1, par 6, ln 5)    **[**My favourite thing was] working hard. I get really puffed out. (Student) (p. 343, clm 1, par 7, ln 5-6) | Unequivocal |
| Create Supportive Therapy Environments: Help kids wade through confusion. (66) | **Participants highlighted the importance of making stuttering a discussable topic in speech therapy, emphasizing the need for children to explore their experiences in a supportive environment. Many participants shared that, as children, they did not have the opportunity to openly discuss their stuttering, leaving them to navigate their confusion on their own. Some participants recalled not initially recognizing their communication difficulties as stuttering, which led to feelings of frustration and uncertainty.** Furthermore, they expressed that, as children, they were unaware of coping strategies for stuttering other than simply trying to get rid of it. ne. Some participants shared that they did not initially recognize their communication challenges as stuttering, leading to frustration and uncertainty. They also expressed that they did not understand that there were options to cope with stuttering beyond trying to get rid of it.  (paraphrased from: p. 105, clm 2, par 4; p. 106, clm 1, par 1-2). | **“We would rather talk about it and then deal with the consequences that come with it than just put it under the rug.” (J.)** (p. 105, clm 2, par 4, ln 4-7)    I had no concept of what stuttering was. I had no idea why I stuttered. I just know that people told me to not do it or to slow down. And the more I tried, the harder it became." (Matthew) (p. 106, clm 1, par 1, ln 1-4)    I think that’s where a lot of the shame comes in. It’s like what is wrong with me? ...I can talk perfectly fine on my own or to a dog. And as soon as it’s someone or in a situation where I’m worried about being judged then I can’t get the words out. (Bill) (p. 106, clm 1, par 1, ln 10-14)    “Kids will find their own solutions to problems if they just start talking about it.” (Jason Jenya) (p. 106, clm 1, par 2, ln 10-11) | Unequivocal |
| Create Supportive Therapy Environments: Validate feelings: It's okay to stutter. (66) | **Participants emphasized the importance of speech therapists being responsive to the social and emotional aspects of stuttering. They highlighted that validating children's feelings and experiences is a crucial part of emotional support. Some participants expressed frustration with others minimizing their struggles and wished for genuine acknowledgment. Instead of having their difficulties dismissed, they wanted to hear that stuttering is okay and to be recognized for their efforts. They also emphasized the importance of reassurance, particularly in helping children understand that they can still achieve their goals while stuttering. Additionally, participants expressed the need for therapists to acknowledge that children are doing their best and to provide encouragement that their way of speaking is okay.** (paraphrased from: p. 106, clm 2, par 2-4) | **“if you don’t know how to help people with [the emotional] part...you’re going to hurt them.” (Rosalinda)** (p.106 clm 2, par 2 ln 3-5)    **“the way to help kids is to let them know that stuttering is okay...to encourage them to speak freely and easily.” (Daisy Duke) (**p.106 clm 2, par 2 ln 5-7)  **“Wow, this is really tough, and this is valid” (Hilton Silver).** (p.106 clm 2, par 3 ln 7-8)    **“It’s so easy, I think, for people or kids to get in their head that idea [that] there’s something fundamentally wrong with them that needs to be fixed” (Owen).** (p.106 clm 2, par 4 ln 3-5)    **“[tell them] they can still be the captain of the tennis team and still stutter. You can still get a girlfriend and stutter, whatever you want.” (Jason Betterman)** (p.106 clm 2, par 4 ln 11-14) | Unequivocal |
| Create safe school environments: Educating others. (66) | **Participants emphasized the need for adults in schools to understand stuttering and provide support. They believed all staff should be educated on the variability of stuttering, the loss of control it involves, and why advice like slow down or take a breath is unhelpful. Educating school staff, parents, and peers was seen as crucial, with suggestions that speech therapists provide training in meetings or one-on-one discussions.** Classroom presentations were also recommended to help peers understand stuttering, but participants stressed that these should be child-driven, allowing the child to decide if and how they want to share their experiences. While presentations could be beneficial, they could also be harmful if not handled with care and without ensuring the child’s autonomy in the process. (paraphrased from: p. 107, clm 2, par 2-3) | **“[showing] there are other people who know and who get it, who [they] can find support in. I think that would be really big” (Felix Felipe).** (p. 107, clm 2, par 2, ln 10-13) | Credible |
| SLPs who work with CWS* should be patient and knowledgeable about stuttering. (67) | **Participants wanted their speech therapists to understand the complexities of stuttering, but often they reported that their speech therapists did not have this knowledge. Participants wanted therapists who were good listeners** and who allowed them to make therapeutic choices based on what has and has not worked for them in the past**.** (p. 7, par 1, ln 1-3) | "I think it’s just try and find out what that person needs by **um listening to them and um what’s um worked for them and um what hasn’t, and um try to be um supportive um even if it’s hard for them"** (CWS 16fc) (p. 7, par 1, ln 1-3)     **"um I would say that you should let the kids who usually stutter a lot more to let them take their time you shouldn’t say it because that’s worse but you should let them talk, you should let them get their words out" (CWS14mc)** (p. 6, Table 4, quote 1)    **“but I never really had a speech therapist who really knew about stuttering and like in the past I’ve had therapists that would come with strategies to like change it or fix it and the thing is that stuttering does not have to be fixed at all.” (CWS16fa)** (p. 6, Table 4, quote 3) | Unequivocal |
| The leadership of the support group: Physiotherapists’ role regarding peer support. (71) | **Previous learners with paraplegia expected the school’s physiotherapist to provide guidance to the peer-group leader by being an approachable source of knowledge, teaching peer supporters, assisting with occasionally giving input and receiving feedback from the group sessions.** In addition, they hoped physiotherapists would advocate for their needs at the school management level **(**p. 7, clm 1, par 2, ln 1-7) | **… there should be the likes of … the help of the hospital physiotherapist, and the school, and other special schools, we will manage.’ (PL2, female, 23 years)** (p.7, clm 1 par 2, ln 8-10)  If one person could go or two persons, and they represent the paraplegics, and they would talk with someone from physio.’ (PL3, female, 26 years) (p.7, clm 1, par 2, ln 11-13)    **They should raise this by someone … maybe, the physio-people.’ (PL3, female, 26 years)** (p. 7,  clm 1, par 2, ln 14-15) | Unequivocal |
| ***Category 14: Understanding and Communicating the Purpose of Therapy (5 findings)***  Children emphasized the importance of understanding why they were in therapy and what it was meant to achieve. While some preferred not to receive detailed information, many valued having clear, accessible explanations about their condition, the goals of therapy, and available treatment options. When therapists took the time to communicate this information effectively, children felt more informed, engaged, and in control of their therapy process. In contrast, some children expressed confusion about the purpose of therapy, which interfered with their ability to make informed treatment choices and diminished their sense of agency | | | |
| Extracted Theme/Category | Verbatim or Paraphrased Description of Extracted Theme/Category | Supporting Illustrations | Level of Credibility |
| Treatment Values: Expectations and emotions around therapy. (Negative) (59) | **One child (participant 4) told that he did not know why he needed to go to the SLP. He explained that he did not know the purpose of the games he played with her.** Nevertheless, he felt that he learned something during the speech and language therapy sessions. When the interviewer followed up with this statement, he could not explain what he was exactly learning. (p. 1532, clm 1, par 1, ln-1-7) | **Participant 4: I don’t know why we do those exercises.**  **Interviewer: But you said that you feel like you are learning something?**  **Participant 4: Yes. I notice that.** (p. 1532, clm 1, par 1, ln-14-17) | Unequivocal |
| Treatment practices: Information flow. (want information) (59) | **Some children valued receiving treatment-related information from SLPs, while others did not. Those who wanted information preferred it to be related to the structure of therapy sessions and the rationale behind therapy exercises.** (paraphrased from: p. 1532, clm 2, par 2, ln 1-5). | **Participant 5: I want to know what we are going to do. And why I need to do it [the exercises].** (p. 1532, par 5, ln 6-7) | Unequivocal |
| Independence. (68) | This theme is used to describe comments participants made about things they are able to do alone and the positive value this has for their well-being (p. 759, par 5, ln 1-2). **However, the notion of independence has also been used to illustrate the lack of awareness some children seemed to have regarding their communication needs. During the groups, particularly within the introduction, participants were asked why they received speech and language therapy. Their responses and comments indicate a lack of awareness of their needs, which may contribute to negative quality of life experiences** (p. 759, par 7, ln 1-6) | **Facilitator: Do you know why you come to speech and language therapy?**  **Male, 6;9: No.**  **Male, 11;1: To be honest I do not actually know.**  (p. 759, par 7, ln 8 -10) | Credible |
| Perceived Relevance of Acquired Knowledge. (70) | **Participants valued the acquisition of knowledge on pain neurophysiology and its contribution to a better understanding of pain.** (p. 198, clm 2 par 3, ln 1-4) | **I think it was interesting and we now have more knowledge on how things work inside our body, and on the processes involved in pain and why we have pain. (Female, group 2)** (p. 198, par 3, ln 5-7) | Unequivocal |
| Perceived Relevance of Acquired Knowledge: Perceived Relevance of Acquired Knowledge as a Complement to Exercise. (70) | **Participants highlight the complementarity between pain neuroscience education and the exercises and the relevance of pain neuroscience education on changing how they perceived exercise and its role in improving their NP.** (p.198, clm 2, par, 4, ln 1-4) | **We have to try and remember everything we learned and say to ourselves: ok, no, this is chronic pain, and I can control it, and do the exercises…**(Female, group 1) (p.198, clm 2, par, 4, ln 5-7) | Unequivocal |

**Note:** The numbers in Column 1 correspond to the study numbers listed in Table 4.  References to the original text use the following abbreviations: *p.* for page, *clm* for column, *par.* for paragraph, and *ln* for line.

**Note**: Bolded text in Columns 2 and 3 highlights the information in the description and illustrations that support the category
